# An Easy-to-Use Plasmid Toolset for Efficient Generation and Benchmarking of Synthetic Small RNAs in Bacteria

**DOI:** 10.1021/acssynbio.2c00164

**Published:** 2022-08-31

**Authors:** Tania S. Köbel, Rafael Melo Palhares, Christin Fromm, Witold Szymanski, Georgia Angelidou, Timo Glatter, Jens Georg, Bork A. Berghoff, Daniel Schindler

**Affiliations:** † RG Schindler, Max-Planck-Institute for Terrestrial Microbiology, Karl-von-Frisch-Street 10, 35043 Marburg, Germany; ‡ MaxGENESYS Biofoundry, Max-Planck-Institute for Terrestrial Microbiology, Karl-von-Frisch-Street 10, 35043 Marburg, Germany; § Institute for Microbiology and Molecular Biology, Justus Liebig University Giessen, Heinrich-Buff-Ring 26-32, 35392 Giessen, Germany; ∥ Mass Spectrometry and Proteomics Core Facility, Max-Planck-Institute for Terrestrial Microbiology, Karl-von-Frisch-Street 10, 35043 Marburg, Germany; ⊥ Institut für Biologie III, Albert-Ludwigs-Universität Freiburg, Schänzlestraße 1, 79104 Freiburg, Germany

**Keywords:** synthetic biology, synthetic sRNA, gene regulation, seed region, sRNA scaffold, antibiotic resistance

## Abstract

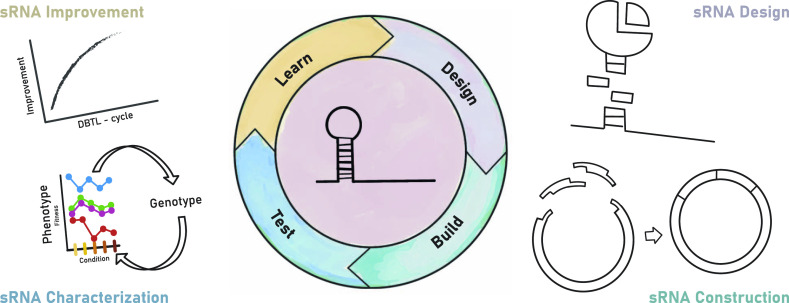

Synthetic biology approaches life from the perspective of an engineer. Standardized and de novo design of genetic parts to subsequently build reproducible and controllable modules, for example, for circuit design, is a key element. To achieve this, natural systems and elements often serve as a blueprint for researchers. Regulation of protein abundance is controlled at DNA, mRNA, and protein levels. Many tools for the activation or repression of transcription or the destabilization of proteins are available, but easy-to-handle minimal regulatory elements on the mRNA level are preferable when translation needs to be modulated. Regulatory RNAs contribute considerably to regulatory networks in all domains of life. In particular, bacteria use small regulatory RNAs (sRNAs) to regulate mRNA translation. Slowly, sRNAs are attracting the interest of using them for broad applications in synthetic biology. Here, we promote a “plug and play” plasmid toolset to quickly and efficiently create synthetic sRNAs to study sRNA biology or their application in bacteria. We propose a simple benchmarking assay by targeting the *acrA* gene of *Escherichia coli* and rendering cells sensitive toward the β-lactam antibiotic oxacillin. We further highlight that it may be necessary to test multiple seed regions and sRNA scaffolds to achieve the desired regulatory effect. The described plasmid toolset allows quick construction and testing of various synthetic sRNAs based on the user’s needs.

## Introduction

Synthetic biology holds the promise to achieve control over gene expression, thereby manipulating phenotypic traits in any organism of interest. Controlling gene expression can be realized at several regulatory layers, for example, by modulating transcription, mRNA stability, or translation initiation. Synthetic regulatory RNAs have emerged as attractive tools for controlling gene expression at the post-transcriptional level in both eukaryotes and prokaryotes.^1,2^ In prokaryotes, post-transcriptional regulation is modulated by RNA-binding proteins (RBPs), *cis*- or *trans*-acting RNAs, and their combination.^3,4^ RBPs mostly act on the global level of mRNA stability and translational processes.^5^ This might be a reason why RBPs are underexplored as post-transcriptional modulators in synthetic biology applications. Recently, bacterial adaptive immune systems, namely, CRISPR–Cas13 and Cas7-11 systems, acting against RNA phages, have been described and are moving into the spotlight as a tool for post-transcriptional control.^6−10^ The application of these CRISPR–Cas systems has so far been mostly limited to eukaryotic cells and cell-free systems. Toxic effects are observed for CRISPR–Cas13, but the Cas7-11 system claims to provide the basis for RNA-targeting tools that are free of off-target and cell toxicity.^9^ Future studies will show whether engineered CRISPR–Cas7-11 systems will have a potential as tools to modulate post-transcriptional processes in prokaryotes despite their large protein size (1,300 to 1,900 amino acids) and the controlled co-expression of CRISPR-RNAs (crRNA).^11^

Besides CRISPR–Cas, *cis*- and *trans*-acting RNAs modulate post-transcriptional processes in prokaryotes. *cis*-Acting RNAs, such as riboswitches or RNA thermometers, form complex secondary structures in the 5′ untranslated region (UTR) of mRNAs and switch their conformation based on a small molecule or an environmental stimulus allowing or preventing mRNA translation. Riboswitches have been explored as tools in synthetic biology for post-transcriptional regulation.^12^ Synthetic riboswitches need to be optimized for a given stimulus. Specificity for synthetic riboswitches can be obtained by the systematic evolution of ligands by the exponential enrichment (SELEX) approach.^13,14^ The resulting riboswitch must be incorporated into the 5′ UTR of the transcript of interest, making them valuable tools as biosensors but cumbersome for large-scale applications in synthetic biology.

**Figure 1:**
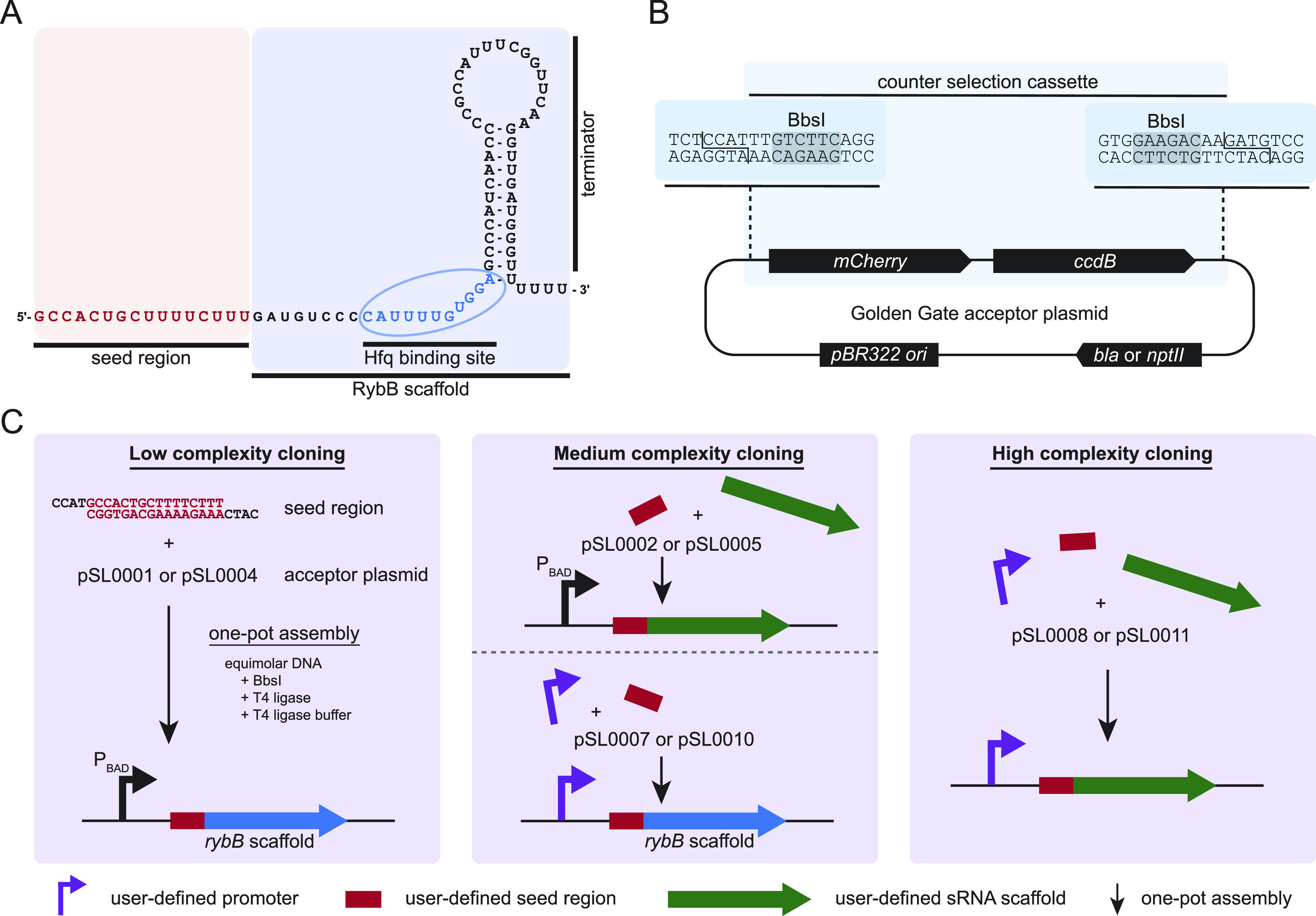
Structure and modules of the sRNA RybB and concept of the plasmid toolset. (A) Modules of wild-type RybB. The wild-type 16-nt seed region (red) consists of an imperfect match, multitargeting antisense sequence. The scaffold of RybB contains two elements, the Hfq binding site and the hairpin structure of the terminator. Structure according to RNAcentral.^40^ (B) Exemplary Golden Gate acceptor plasmid [pSL0001 (Amp^R^) or pSL0004 (Kan^R^)] highlights the counter selection cassette containing the *mCherry* and *ccdB* gene flanked by BbsI recognition sites (highlighted in gray) to facilitate efficient type IIS-based cloning. The cloning relies on the type IIS recognition sites which are lost from the plasmid if the desired insert is ligated into the plasmid backbone, allowing a single-step, one-pot reaction.^35^ All constructed accepter vectors of the toolset contain the same counter selection cassette only differing in the sequence of the restriction sites and the properties of the plasmid backbone, allowing different complexities of cloning. (C) Different levels of cloning complexity that can be performed with the toolset. The low-complexity cloning allows the integration of any designed seed region into acceptor plasmids, resulting in a synthetic RybB TU under P_BAD_ control (left panel). The medium-complexity cloning allows either the cloning of a designed seed region and an sRNA scaffold or the cloning of a promoter of choice and a seed region (center panel). The high-complexity cloning allows the user the combination of multiple fragments to create a synthetic sRNA TU. Visualized are the combination of the promoter, seed region, and sRNA scaffold, but more fragments could be assembled if the matching overhangs are designed (right panel).

In recent years, *trans*-acting small RNAs (sRNAs) have gained attention because their in-depth characterization has revealed many underlying features that enable the relatively easy design of customized synthetic sRNAs.^15−18^ Importantly, most sRNAs have a size of <100 nucleotides (nt), simplifying assembly strategies and reducing the costs for DNA synthesis. Another advantage is their modular structure, which allows adjustment of the sRNA regulator to the desired applications. Prototype sRNAs consist of two building blocks: the seed region and the scaffold,^19^ as exemplified by the well-studied sRNA RybB. In *Escherichia coli*, RybB consists of the 16-nt seed region at its 5′ end and the 63-nt scaffold (Figure 1A). The seed region is the “regulatory module” that is sufficient for target regulation,^20,21^ and the scaffold is the “structural module” that enables binding to the important RNA chaperone Hfq, which in turn supports RNA–RNA interactions.^22−24^ In many cases, seed regions bind to the translation initiation region (TIR), consisting of the Shine–Dalgarno (SD) sequence and the start codon, to block translation.^19,25^ However, translational repression might also occur through binding within the 5′ UTR upstream of the TIR^26,27^ or within a “five codon window” of the coding region,^21^ and mRNA destabilization can be initiated by targeting the coding region.^28^ In contrast to natural sRNAs that regulate multiple targets and only show limited complementarity to each individual target, seed regions of synthetic sRNAs are designed to be fully complementary to one selected target mRNA. While selection of the seed region and scaffold represents a crucial step in synthetic sRNA design, the regulatory outcome can be modulated further by the sRNA expression strength.^17,29^ All these considerations have paved the way for phenotypic modulation of bacteria and biotechnological applications using synthetic sRNAs.^29−31^

Here, we present an easy-to-use and efficient resource to quickly create synthetic sRNA expression constructs by the use of Golden Gate cloning. The system can be applied by any molecular biology laboratory without the need for extensive resources and part libraries allowing “plug and play” synthetic biology. The *E. coli* sRNA RybB is used as an exemplary model to characterize the system. Constructed synthetic sRNAs are benchmarked using a simple readout by altering *E. coli* cells sensitive to the β-lactam antibiotic oxacillin. After an initial screen, in-depth characterization of two candidates is performed. We further transfer our concept to other sRNAs, namely, MicA, MicF, and OmrB. Our results are in line with other research showing that the expression and regulatory impact of synthetic sRNAs can be fine-tuned based on the combination of the promoter, seed region, and sRNA scaffold. The presented cloning systems for synthetic sRNA generation are expected to be highly valuable tools for basic and applied sciences.

## Results and Discussion

### Modular Toolbox for Rapid Synthetic sRNA Construction

Proof-of-concept studies have shown that sRNAs can be modularized according to synthetic biology design principles to (i) study their function and (ii) to create synthetic sRNAs as specific regulators.^31,32^ Throughout this study, the well-characterized sRNA RybB (Figure 1A) was used as a model system, but the concept can be expanded to any other sRNA. RybB was chosen as a prototype sRNA that has already proven useful for the design of synthetic sRNAs and phenotypic screens in different gammaproteobacteria.^20,33^ To allow systematic characterization and application of sRNAs, a set of pBAD plasmid derivatives was constructed for fast and efficient Golden Gate cloning. The aim is to have a simple and reliable system that can be applied in any molecular biology laboratory to make the first steps toward applying synthetic biology concepts. Golden Gate cloning is based on type IIS restriction enzymes which have a directed recognition site and cut any sequence in a defined distance.^34^ By designing matching DNA overhangs, multiple DNA fragments can be assembled simultaneously in a single reaction.^35^ The fragments have the requirements of being double-stranded DNA, containing no internal recognition sequences for the type IIS restriction enzyme(s) being used and being equipped with matching overhangs. The pBAD plasmid was selected to construct acceptor plasmids based on its well characterized properties and the absence of BbsI (isoschizomers: BpiI, BpuAI, and BstV2I) recognition sites.^36^ For this reason, BbsI was chosen for cloning of sRNA transcriptional units (TUs) (Figure 1B). To allow highly efficient TU-cloning, a dual selection cassette was created and used to construct derivatives of the pBAD plasmid. The selection cassette contains genes for a red fluorescent protein (*mCherry*) and a toxin (*ccdB*, encoding a gyrase inhibitor). The resulting acceptor plasmids can only be propagated in the respective *E. coli* strains containing gyrase mutations or containing the *ccdA* antidote.^37,38^ This dual selection cassette was adjusted based on Schindler et al. (2016) where *lacZ*α is used as a visual indicator. Changing *lacZ*α to a fluorescence marker avoids the use of X-Gal (5-bromo-4-chloro-3-indolyl-β-d-galactopyranoside) but still allows visual identification of clones escaping the counter-selection pressure (*ccdB* or gyrase-associated mutations) in case high-efficiency cloning is compulsory (i.e., for combinatorial or library cloning).^39^*mCherry* is under control of a P_lac_ promoter preventing high expression and allowing induction if necessary. Transformation after Golden Gate cloning reactions is performed into *ccdB*-sensitive *E. coli* cells (here *E. coli* TOP10 or MG1655). Only cells which received assembled plasmids (loss of dual selection marker cassette) can grow, resulting in highly efficient DNA assembly.

Four different types of acceptor plasmids were generated by Gibson assembly^41^ with ampicillin (β-lactamase, *bla*) and kanamycin (neomycin phosphatase, *npt*II) resistance markers, resulting in eight acceptor plasmids (Table 2). Each acceptor plasmid type allows different levels of complexity to assemble sRNA TUs (Figure 1C). The lowest complexity level allows cloning of designed seed regions to construct synthetic RybB derivatives under control of the l-arabinose-inducible P_BAD_ promoter (pSL0001 and pSL0004). The seed regions can be obtained as forward and reverse primer pairs containing the matching overhangs for the Golden Gate cloning (cf. Supporting Information, Table S1). The medium-complexity level contains two types of plasmids allowing either the simultaneous assembly of the seed region and an sRNA scaffold downstream of the P_BAD_ promoter (pSL0002 and pSL0005) or promoter and seed region assembly upstream of the RybB scaffold (pSL0007 and pSL0010). The promoter or sRNA sequences can be generated by PCR with designed sequences attached to the amplification sequence of the primer pairs encoding the compulsory BbsI recognition and cut sites. Alternatively, the same fragment can be obtained by gene synthesis which may have an advantage to prevent internal type IIS recognition sites and gives the full control of the DNA sequence. In case only short sequences are required, they can be ordered as primer pairs containing the matching overhangs after annealing. However, in this case, it is necessary to phosphorylate at least one of the annealed primer pairs to allow efficient ligation within the reaction. The fourth type of plasmids (pSL0008 and pSL0011) gives the maximum flexibility and is intended to combine the promoter, seed region, and sRNA scaffold but would allow the combination of more fragments if needed (cf.^42−44^ for overhang evaluation). Within this study, only pSL0004, pSL0010, and pSL0011 are used; however, all plasmids are freely available upon request.

The constructed plasmids can be used in standard manual cloning procedures in any molecular biology laboratory. The efficient cloning allows the quick creation of new combinations and their characterization in the desired host strain. Besides this, the set of plasmids can easily be used in high-throughput cloning strategies harnessing the power of laboratory automation. In this study, the assembly of 48 plasmids (detailed in the next section, not all data shown) was miniaturized to a total reaction volume of 1 μL each with an acoustic dispenser (Echo525, Labcyte) resulting in an approximately 20-fold cost reduction on reagents. After transformation and selection, two candidates for each reaction were analyzed by colony PCR, resulting in 96.9% being the expected construct. We took only two colonies for each transformation because we observed in our initial test of four Golden Gate reactions that all but one single clone screened by colony PCR had the correct insert out of 96 candidates (4 × 24 candidates). The colony PCR used the respective seed region-specific primer as a forward primer and a universal reverse primer binding in the plasmid backbone. We obtained, on average, 2138 ± 738 candidates per transformation of our 1 μL reaction with the described setup and cloning strategy. Extraction of plasmids was performed in the 96-well format based on an open source magnetic bead procedure.^45^ Subsequent transfer of plasmids into desired strain backgrounds for further characterization was performed by the transformation and storage solution (TSS) method.^46^ The workflow is economical, reproducible, and highly scalable. The cloning, validation, and transfer workflow can be completed within 4 days, allowing commencement of characterization of candidates on the fourth day, even without the need for laboratory automation. The introduced plasmid set creates a modular toolbox for rapid synthetic sRNA construction with various levels of complexity based on the user’s needs.

### Benchmarking Synthetic sRNA Functionality by Rendering *E. coli* Cells Sensitive toward the β-Lactam Antibiotic Oxacillin

**Figure 2:**
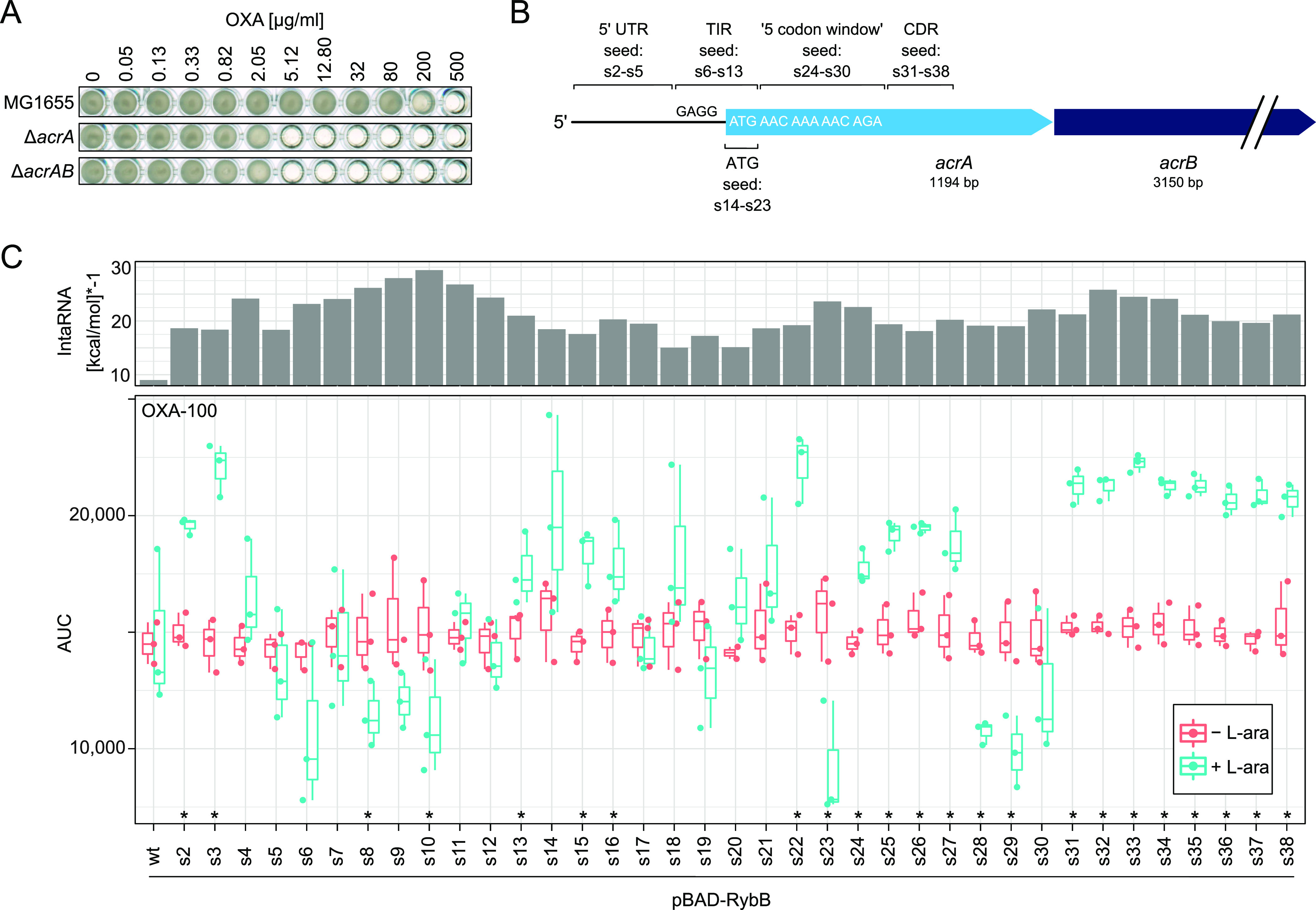
Phenotypic screening identifies seed regions for efficient *acrA* targeting. (A) MIC determination for *acrA* and *acrAB* deletion strains. Stationary-phase cultures were diluted 1,000-fold and loaded into 96-well plates. Oxacillin (OXA) was present at the indicated concentrations (2.5-fold dilution series starting at 500 μg/mL). A well without oxacillin was used as growth control. The 96-well plates were incubated at 37 °C under continuous shaking for 24 h. A representative experiment is shown. (B) Schematic representation of the *acrAB* operon. The relevant binding regions of seed regions s2–s38 are indicated. “GAGG” represents the *acrA* SD sequence. The first five *acrA* codons are given. (5′ UTR: 5′ untranslated region; TIR: translation initiation region; ATG: start codon; and CDR: coding region). (C) Phenotypic screening of synthetic RybB sRNAs with seed regions s2–s38. Seed regions were cloned into the pBAD derivative pSL0004 for inducible expression of synthetic RybB sRNAs. Stationary-phase cultures were inoculated in 96-well plates to monitor growth (OD_600_) in a plate reader. LB medium contained oxacillin at 100 μg/mL (OXA-100). Strains were treated with l-arabinose (+l-ara) to induce sRNA expression or left untreated (−l-ara). The growth was assessed by calculating the AUCs. Data of individual biological replicates (dots) were combined and illustrated as boxplots (*n* = 3, except for s20 without l-ara: *n* = 2). Student’s *t*-test was applied for statistical testing (*: *P* < 0.05). The histogram on the top indicates binding energies for sRNA–*acrA* pairs, as calculated by IntaRNA.^53^ Negative binding energies were multiplied by −1 for illustrative purposes. Wild-type (wt) RybB is shown for comparison.

Synthetic sRNAs can be applied for regulating almost every gene of interest.^47^ Selection of the seed region is a critical step in synthetic sRNA design.^15^ However, the regulation strength of a distinct seed region might be difficult to predict, which suggests that experimental testing of numerous seed regions is beneficial to identify the best seed region for regulation. We followed such a strategy and made use of the pBAD derivative pSL0004 for fast and efficient low-complexity cloning of synthetic RybB sRNAs with variable seed regions (Figure 1C). As the target gene, we selected *acrA*, which is part of the *acrAB* operon and encodes a membrane fusion lipoprotein of the AcrAB–TolC multidrug efflux pump.^48^ Deletion of the *acrAB* operon is known to increase the susceptibility to different antibiotics, including the β-lactam antibiotic oxacillin.^49^ In *E. coli* MG1655, both an *acrA* deletion alone and an *acrAB* operon deletion were sufficient to decrease the minimum inhibitory concentration (MIC) of oxacillin to 5.12 μg/mL, which is an ∼100-fold decrease in comparison to the wild type (MIC of 500 μg/mL; Figure 2A). The *acrA* gene represents an ideal target for sRNA-based regulation,^50^ and the oxacillin-susceptible phenotype enables fast evaluation of the regulatory potential of different seed regions. We designed 38 different seed regions, each with a length of 16 nt, and fused them to the RybB scaffold sequence on plasmid pSL0004 to obtain sRNA expression plasmids. The multi-targeting wild-type RybB seed region was used as a control, as it does not target *acrA*. The remaining 37 seed regions were designed in such a way that they covered important regulatory regions of the *acrA* mRNA. We refer to these seed regions using the s-numbers s2 to s38 (Figure 2B). For targeting of the 5′ UTR, four seed regions (s2–s5) were designed in a nonoverlapping fashion, covering almost the complete 5′ UTR from the transcriptional start site to the SD sequence. For targeting of the TIR, start codon and “five codon window”, 25 seed regions were designed in a staggered fashion using a shift of one nucleotide. We designed eight seed regions for the TIR (s6–s13), ten seed regions for the start codon (s14–s23), and seven seed regions for the “five codon window” (s24–s30). The coding region following the “five codon window” was covered by eight nonoverlapping seed regions (s31–s38). A detailed illustration of the seed region design is shown in the Supporting Information (Figure S1). Secondary structure predictions, using RNAfold from the Vienna RNA websuite,^51^ indicated that none of the synthetic seed regions affected the integrity of the terminator hairpin (nucleotides 34–79). In contrast, internal base-pair probabilities within the first 33 nucleotides were clearly different, giving rise to single-stranded seed regions and seed regions that were mainly occluded in stem-loop structures (Supporting Information, Figure S2). To assess functionality of the synthetic RybB sRNAs, the *acrA*-dependent oxacillin susceptibility was evaluated. We realized, however, that a regular MIC test (as shown in Figure 2A) is not suitable because it is based on endpoint measurements of the optical density at 24 h after inoculation. Endpoint measurements are easily confounded by suppressor mutants. These mutants can have the same oxacillin susceptibility as the wild type and easily overgrow the remaining population. In line with this scenario, we observed that selected sRNA expression strains produced high optical densities even at 200 μg/mL oxacillin (data not shown), which was comparable to the wild type (Figure 2A). We therefore monitored the growth of sRNA expression strains in a plate reader, immediately starting at the time point of inoculation. In an initial screening experiment, strains were cultivated in lysogeny broth (LB) medium with and without the inducer l-arabinose at different oxacillin concentrations (0–200 μg/mL; Supporting Information, Figure S3A). The addition of β-lactam antibiotics causes filamentation of *E. coli*,^52^ and the same was observed here when using oxacillin (data not shown). The irregular shape of the resulting growth curves impeded calculation of common growth parameters (Supporting Information, Figure S3B), and therefore, the areas under the curves (AUCs) were calculated to assess differences in growth. Log_2_ fold-changes indicate the ratio of AUC values from l-arabinose-treated and untreated cultures (+/– l-ara). In LB medium without oxacillin, the addition of l-arabinose promoted growth of all strains, as indicated by log_2_ fold-changes of 0.15–0.76 (Supporting Information, Figure S3A). As soon as oxacillin was present, several strains with sRNA expression plasmids were inhibited in their growth (log_2_ fold-changes < 0), and for these, the general trend emerged that log_2_ fold-changes decreased with increasing oxacillin concentrations. In contrast, the growth of strains containing the empty control plasmid pSL0003 or expressing wild-type RybB was still promoted or at least unaffected by l-arabinose, even at the highest oxacillin concentration (200 μg/mL; Supporting Information, Figure S3A). Collectively, these observations suggested that several of the tested synthetic RybB sRNAs caused an increase in susceptibility to oxacillin through repression of *acrA* mRNA. Since the initial screening experiment revealed that an oxacillin concentration of 100 μg/mL was well suited to observe sRNA effects, we repeated the experiment using a concentration of 100 μg/mL (Figure 2C). We compared our experimental data to computational data and used the IntaRNA tool to predict the binding energy for all sRNA–*acrA* interactions.^53^ As expected, wild-type RybB had the highest binding energy of −9.01 kcal/mol, indicating poor binding to the *acrA* mRNA (for illustrative purposes, binding energies were multiplied by −1 in Figure 2C). In contrast, all *acrA*-targeting sRNAs had lower binding energies ranging from −15.03 (RybB-s18) to −29.45 kcal/mol (RybB-s10; Figure 2C). There was no clear-cut match between experimental and prediction data, except for seed regions that targeted the *acrA* TIR (s6–s13), as supported by a positive correlation between energy values and log_2_ fold-changes at 100 μg/mL oxacillin (Pearson’s *r* of 0.6; Supporting Information, Figure S3C). Seed regions that targeted the *acrA* coding region (s31–s38) were not effective in growth inhibition, even though some of the corresponding sRNAs had fairly low predicted binding energies (e.g., RybB-s32: −25.81 kcal/mol; Figure 2C). These findings clearly underscore the notion that most efficient regulation is achieved by sRNA binding in close proximity to the TIR.^54^ Furthermore, our data show that the experimental screening approach is especially useful to identify regulatory hotspots, as in the case of *acrA* validated for seed regions s6–s10 (TIR), s23 (start codon), and s28–s29 (“five codon window”). Importantly, sRNAs RybB-s28 and RybB-s29 would have been missed by the computational approach, since both sRNAs had comparably high binding energies of −19.12 and −19.02 kcal/mol, respectively.

### Detailed Analysis of *acrA* Regulation by RybB-s8 and RybB-s38 Using a Constitutive Expression System

**Figure 3:**
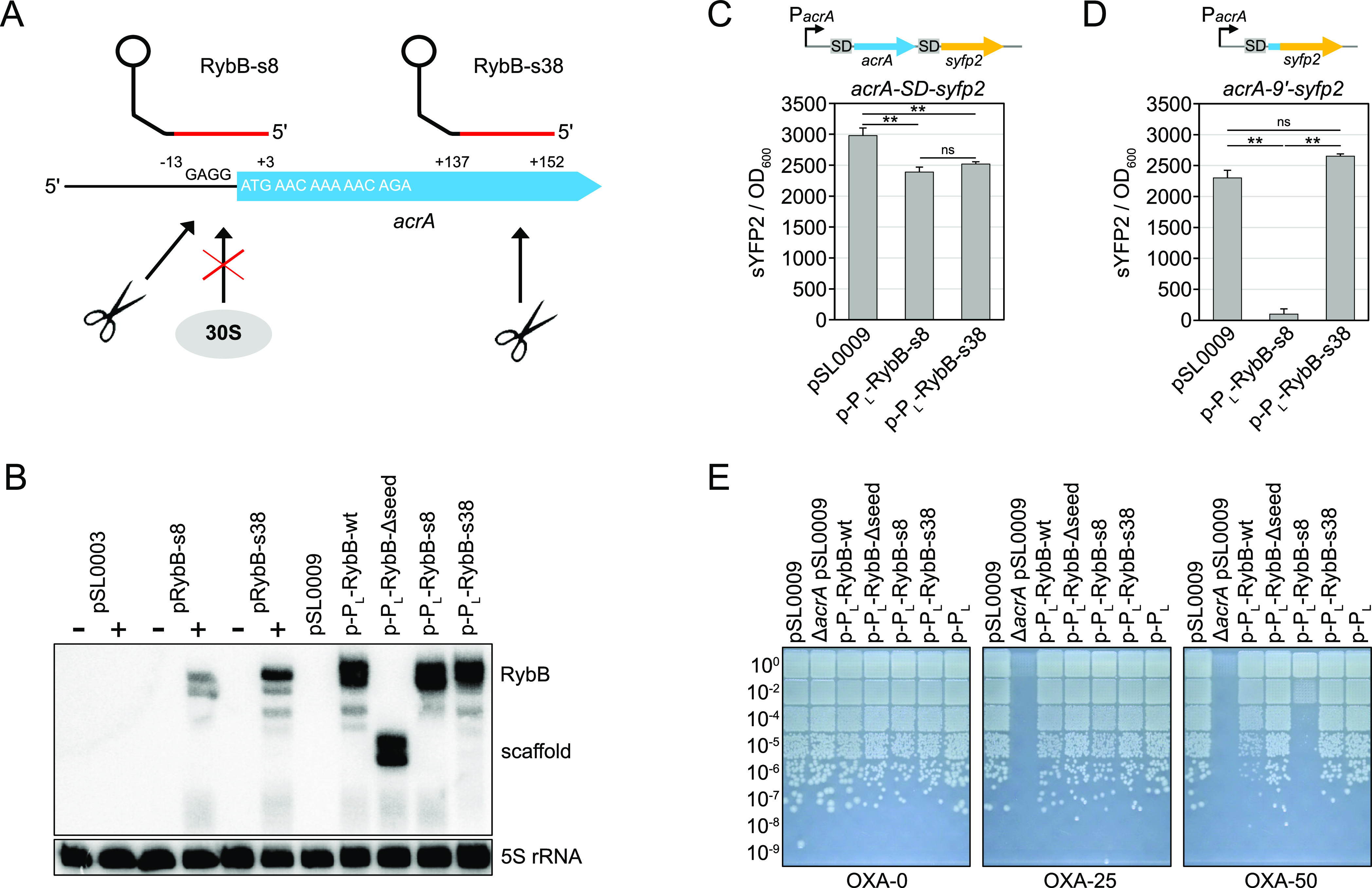
Constitutive expression of the *acrA*-targeting sRNA RybB-s8 increases the susceptibility to oxacillin. (A) Schematic representation of sRNA binding sites. Seed regions of synthetic RybB sRNAs are highlighted in red. Their binding positions on the *acrA* mRNA refer to the “A” of the start codon (position +1). The RybB terminator hairpin is shown as a lollipop structure. “GAGG” represents the *acrA* SD sequence. The first five *acrA* codons are given. The potential regulatory outcome is either hindrance of translation initiation by the 30S ribosomal subunit or initiation of mRNA degradation by RNases (scissors). (B) Northern blot analysis of sRNA expression strains. Strains containing the empty control plasmid pSL0003 or pBAD expression plasmids pRybB-s8 and pRybB-s38 were grown to the exponential phase. Samples were withdrawn before (−) and 30 min after the addition of l-arabinose (+). For constitutive sRNA expression experiments using the P_L_lacO-1 promoter (p-P_L_ plasmids), samples were withdrawn from exponential-phase cultures. pSL0009 served as an empty control plasmid. A radioactive probe targeting the RybB scaffold sequence was used for detection of RybB sRNAs. 5S rRNA was probed as a loading control. (C,D) Effect of sRNA expression on sYFP2 fluorescence from chromosomal *acrA* reporter constructs. The constructs are illustrated on top of the graphs. The transcriptional reporter *acrA*–*SD*–*syfp2* (C) is transcribed from the native *acrA* promoter (P*acrA*) and represents a fusion of the complete *acrA* (blue) and *syfp2* (yellow) open reading frames, each preceded by an SD sequence. The translational reporter *acrA*-9′–*syfp2* (D) is transcribed from the native *acrA* promoter (P*acrA*) and represents a fusion of the first nine *acrA* codons (blue) to the *syfp2* (yellow) open reading frame, lacking its own start codon. Reporter strains either contained empty control plasmid pSL0009 or sRNA expression plasmids p-P_L_-RybB-s8 and p-P_L_-RybB-s38. Stationary-phase cultures were diluted 100-fold into LB medium, and sYFP2 fluorescence (excitation: 510 nm and emission: 540 nm) and OD_600_ were measured after 6 h of cultivation in a microplate reader. Values were background-corrected, and sYFP2 fluorescence was normalized to the OD_600_. The mean of three independent biological replicates is shown. Error bars represent the standard deviation. One-way ANOVA with post-hoc Tukey HSD was applied for statistical testing (**: *P* < 0.01 and ns: not significant). (E) Oxacillin susceptibility assay. Stationary-phase cultures were serially diluted as indicated and spotted onto LB agar plates containing varying concentrations of oxacillin (OXA, 0–50 μg/mL). Plates were incubated overnight at 37 °C. The empty plasmid pSL0009, wild-type RybB (RybB-wt), RybB lacking a seed region (RybB-Δseed), and a plasmid containing the P_L_lacO-1 promoter (p-P_L_) were used as controls. Representative results are shown.

We selected two synthetic RybB sRNAs from the initial screening approach for further analysis. RybB-s8 was selected because its seed region covers the entire TIR (Figure 3A and Supporting Information, Figure S1), and it was consistently scored with low log_2_ fold-changes in the presence of oxacillin (Figure 2C and Supporting Information, Figure S3A). Since RybB-s8 binds to the TIR, it is expected to block translation initiation and probably destabilize *acrA* mRNA. RybB-s38 was selected as the second candidate. The seed region of RybB-s38 is targeted toward the *acrA* coding region (position +137–152 with respect to the start codon; Figure 3A and Supporting Information, Figure S1), and RybB-s38 might therefore destabilize *acrA* mRNA to increase oxacillin susceptibility. However, RybB-s38 only scored a slightly negative log_2_ fold-change (−0.17) at the highest oxacillin concentration (200 μg/mL) during the initial screening approach (Supporting Information, Figure S3A). In order to increase the regulatory potential of both synthetic sRNAs, we made use of cloning plasmid pSL0010 (Figure 1C) for single-step assembly of (i) a promoter sequence, (ii) a seed region, and (iii) the RybB scaffold-containing plasmid backbone. We used the P_L_lacO-1 promoter to obtain constitutive expression of sRNAs.^28,55^ To evaluate expression of the synthetic sRNAs and to compare the different expression systems, Northern blot analysis was performed in an *E. coli* MG1655 *rybB* deletion background. As expected, RybB was not detectable in strains containing empty control plasmids pSL0003 and pSL0009 (Figure 3B). In strains containing pBAD plasmids, RybB-s8 and RybB-s38 were only detected after induction with l-arabinose. In contrast, using the P_L_lacO-1 promoter resulted in constitutive sRNA expression that was, on average, approximately twofold stronger than with the inducible pBAD system (Figure 3B). To evaluate the regulatory effect of the constitutively expressed sRNAs on *acrA*, we constructed *syfp2* reporter fusions in the MG1655 chromosome. Transcriptional (*acrA*–*SD*–*syfp2*) and translational (*acrA*-9′–*syfp2*) fusions were used to discriminate between sRNA effects on mRNA stability and translation initiation, respectively. Both RybB-s8 and RybB-s38 significantly decreased sYFP2 fluorescence from the transcriptional fusion by 1.2-fold in comparison to the control (Figure 3C), suggesting a slight negative effect on mRNA stability. However, RybB-s38 did not affect fluorescence from the translational fusion, which was expected because RybB-s38 cannot bind to the *acrA* TIR to block translation. RybB-s8, on the other hand, caused a strong decrease (22.9-fold) in fluorescence from the translational fusion (Figure 3D), which is in accordance with blocking translation initiation due to binding to the *acrA* TIR. Strong translational repression of *acrA* is expected to decrease AcrA protein amounts and, consequently, increase the oxacillin susceptibility. To assess the oxacillin susceptibility, stationary-phase cultures were serially diluted and spotted on LB agar plates containing varying amounts of oxacillin. As controls, plasmids for constitutive expression of RybB either containing its native seed region (RybB-wt) or lacking a seed region (RybB-Δseed) were constructed and validated by Northern blot analysis (Figure 3B). Finally, the *rybB* sequence was omitted altogether to evaluate the effect of the P_L_lacO-1 promoter alone (plasmid p-P_L_). All strains showed a similar growth on LB agar plates without oxacillin (Figure 3E). At 25 μg/mL oxacillin, only the *acrA* deletion strain did not grow, which was in agreement with an MIC of 5.12 μg/mL (Figure 2A). At 50 μg/mL oxacillin, constitutive expression of RybB-s8 caused a clear growth defect, which was not observed for RybB-s38 or the control plasmids (Figure 3E). RybB-s38 failed to cause a growth defect because it was not able to block translation (Figure 3D) and, consequently, was not able to lower AcrA protein amounts, as also confirmed by proteome analysis (see below). Together, these data indicate that strong translational repression of *acrA* by RybB-s8 is sufficient to increase oxacillin susceptibility and that the s8 seed region is the single cause for this phenotype. We note, however, that expression of wild-type RybB affects growth at higher oxacillin concentrations (Supporting Information, Figure S4). The seed region of wild-type RybB binds to the 5′ UTR of *csgD*, encoding the master regulator for production of curli fibers in *E. coli*, thereby interfering with *csgD* expression. Since curli are important components of the biofilm matrix that physically protects bacteria from antibiotics, a reduced production of curli upon overexpression of wild-type RybB is a likely explanation for the observed oxacillin sensitivity at higher concentrations.^56^

### Proteome Analysis Validates that Synthetic RybB-s8 sRNA Alters AcrA Abundance

**Figure 4:**
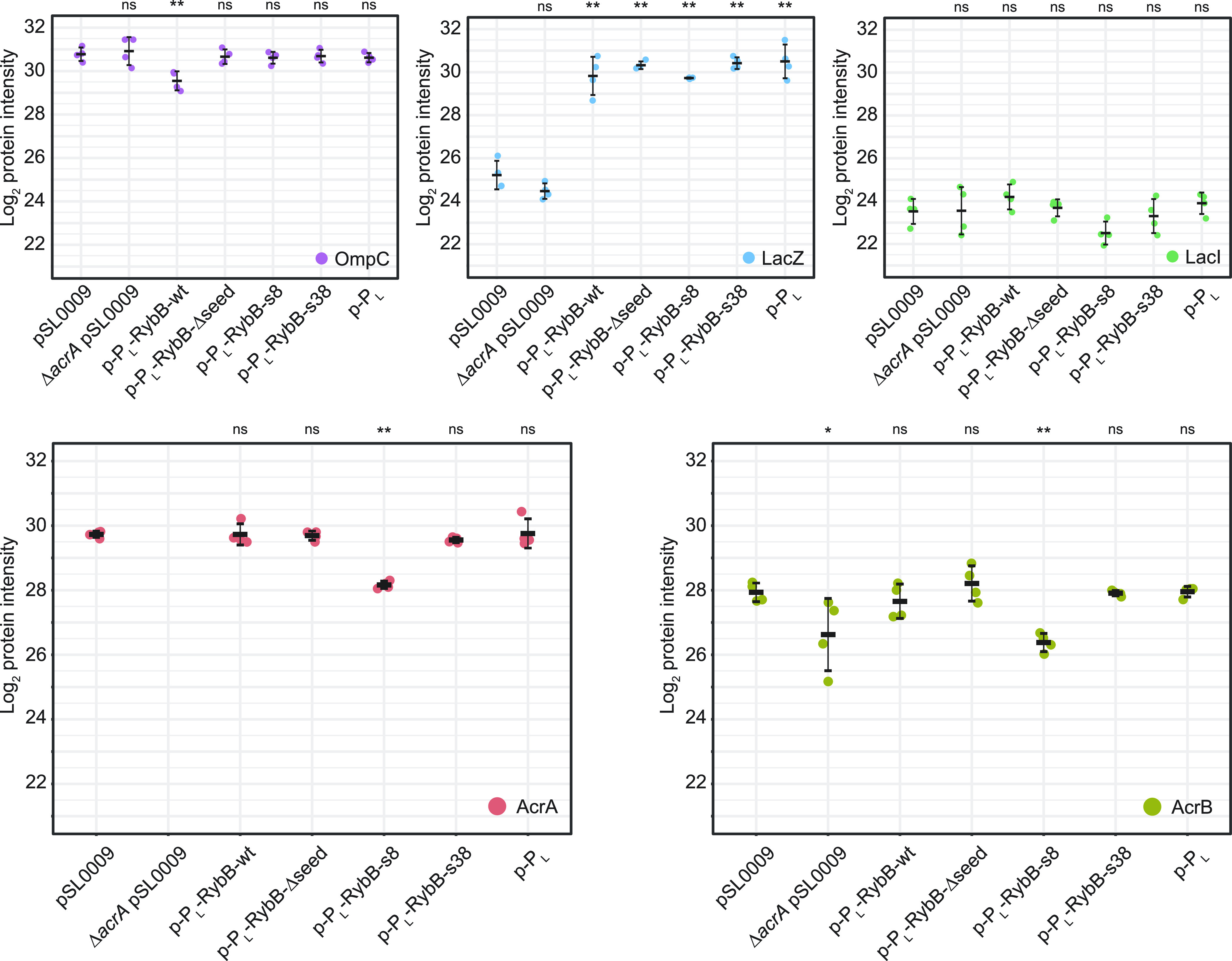
Determination of protein abundances by mass spectrometry. Proteome analysis of strains containing p-P_L_-RybB-s8 and p-P_L_-RybB-s38 was performed in comparison to p-P_L_-RybB-wt and additional control strains in quadruplicates in the absence of oxacillin. Whisker plots show the median, minimum, and maximum of log_2_ intensities for the respective protein, while dots indicate individual measurements. OmpC is one of the native targets of RybB-wt and significantly reduced if RybB-wt is expressed. If the seed region is removed (RybB-Δseed) or altered (RybB-s8 and RybB-s38), this regulation is abrogated. While analyzing the data, a significant increase in LacZ was observed for constructs containing promoter P_L_lacO-1, which can be explained by LacI titration via the additional *lac*-operator sequences in the used P_L_lacO-1 promoter. The *lac*-repressor LacI shows no alteration in abundance supporting this hypothesis. RybB-s8 expression reduces the abundance of AcrA and AcrB. RybB-s38 expression does not reduce AcrA and AcrB abundances. One-way ANOVA with post-hoc Tukey HSD was applied for statistical testing. Significance levels indicate the comparison to empty plasmid pSL0009 (*: *P* < 0.05, **: *P* < 0.01, and ns: not significant).

To further investigate the influence of sRNA expression on protein abundances, whole proteome analysis was performed. Figure 4 shows the protein intensities from a mass spectrometry analysis to visualize relevant protein abundances (see the Materials and Methods section for details). Expression of wild-type RybB showed a clear downregulation of the native target OmpC (Figure 4), while OmpC had a normal abundance if the natural seed region of RybB was not present or replaced with a synthetic seed region. These results indicate that the applied RybB scaffold is lacking its native function and support the assumption that the scaffold can serve as a backbone for synthetic sRNAs.^20,33^ Interestingly, all strains containing the P_L_lacO-1 promoter showed a higher abundance of LacZ, which is encoded in the LacI-controlled *lac* operon. However, the abundance of LacI did not change, suggesting that the higher abundance of LacZ was a direct cause of LacI titration by the additional repressor binding sites in plasmids containing the P_L_lacO-1 promoter (the sequence results in approximately 30–40 additional sites based on the previously described copy number of ∼15–20 for plasmids with pBR322 origin of replication).^57^ Accordingly, the samples were quantified against the strain containing the p-P_L_ plasmid to quantify the change in AcrA abundance in response to RybB-s8 and RybB-s38 expression. AcrA abundance was approximately threefold reduced as a result of RybB-s8 expression (Figure 4), corroborating the results from our sYFP2 reporter assays (Figure 3D) and oxacillin susceptibility tests (Figure 3E). AcrB, encoded by the second gene in the *acrAB* operon (Figure 2B), had an ∼4.5-fold reduction, indicating translational coupling of *acrA* and *acrB* as no effect was observed for the transcriptional reporter fusion (Figure 3C). In contrast to RybB-s8, expression of RybB-s38 did not alter AcrA or AcrB abundance. Strikingly, neither expression of wt-RybB nor RybB-Δseed showed an alteration of the AcrA or AcrB protein levels, further underscoring that synthetic RybB sRNAs can be powerful and reliable tools for manipulation of gene expression.^31,32^

### Validation of Alternative sRNA Scaffolds Using the High-Complexity Cloning System

**Figure 5:**
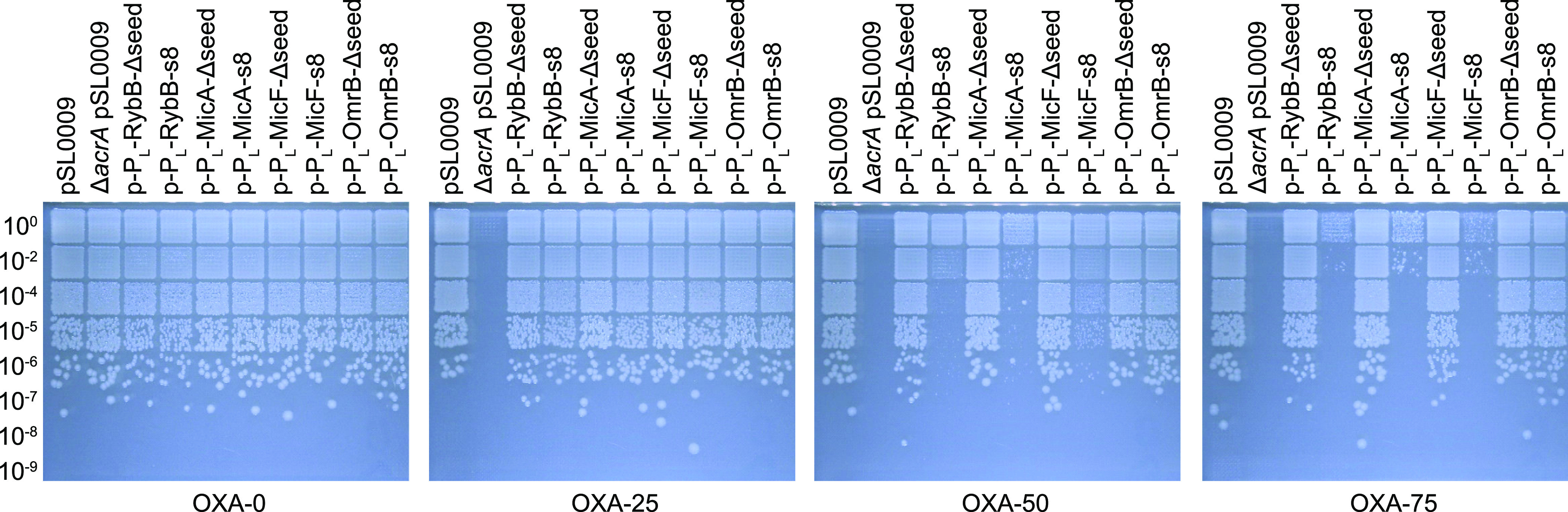
Evaluation of alternative sRNA scaffolds. Oxacillin susceptibility assays were performed upon expression of the synthetic sRNAs MicA-s8, MicF-s8, and OmrB-s8. Stationary-phase cultures were serially diluted as indicated and spotted onto LB agar plates containing varying concentrations of oxacillin (OXA, 0–75 μg/mL). Plates were incubated overnight at 37 °C before images were acquired. The empty plasmid pSL0009 and sRNAs lacking a seed region (Δseed) serve as controls. Representative results are shown.

After confirmation that the RybB-s8 construct was responsible for increased oxacillin susceptibility, we asked the question whether fusion of the s8 seed region to alternative sRNA scaffolds would produce similar results. It is known from other studies that seed regions can be successfully transplanted to alternative scaffolds.^20,31^ We selected the sRNAs MicA, MicF, and OmrB because they are well-studied in *E. coli* and—like RybB—contain seed regions at their 5′ ends (Supporting Information, Figure S5). We note, however, that several other sRNAs exist that might be useful as starting points for synthetic sRNA design.^15,30,31^ We made use of cloning plasmid pSL0011 (Figure 1C) to generate constructs with (i) the P_L_lacO-1 promoter, (ii) the s8 seed region, and (iii) the aforementioned alternative sRNA scaffolds. To exclude the possibility that the newly selected scaffolds affect oxacillin susceptibility by themselves, variants without a seed region were constructed as well (Δseed). To evaluate oxacillin susceptibility, solid media dilution assays were performed as before (cf. Figure 3E). As seen for RybB-s8, at 50 μg/mL oxacillin, constitutive expression of MicA-s8 and MicF-s8 also caused an increased susceptibility, albeit the effect was less pronounced for MicF-s8 (Figure 5). At an oxacillin concentration of 75 μg/mL, however, RybB-s8, MicA-s8, and MicF-s8 performed comparably. These results corroborate that the s8 seed region can be functionally fused to different sRNA scaffolds. In the case of OmrB-s8, a slight inhibitory effect was only observed at higher concentrations (125 μg/mL oxacillin; Supporting Information, Figure S6). A possible explanation for the weak performance of OmrB-s8 might be found in its predicted secondary structure. Both RNAfold^51^ and Mfold^58^ predict that the s8 seed region is almost completely occluded in a stem-loop structure formed at the 5′ end of OmrB-s8 (Supporting Information, Figures S7 and S8). This possibly renders the s8 seed region inaccessible for base-pairing with *acrA* mRNA, which is not the case with MicA-s8 and MicF-s8. This highlights the importance not only of identifying suitable seed-sequences but furthermore that the selected seed-sequence must match the chosen sRNA scaffold. Another interesting observation concerns the MicF scaffold (MicF-Δseed). At an oxacillin concentration of 100 μg/mL and above, the MicF scaffold alone caused a growth defect (Supporting Information, Figure S6). The P_L_lacO-1 promoter used is a strong constitutive promoter, likely causing elevated levels of the MicF scaffold, as also observed for the RybB scaffold (Figure 3B). Since secondary structure predictions indicate that the MicF scaffold has 31 unpaired nucleotides at its 5′ end (Supporting Information, Figures S7 and S8), we assume that the MicF scaffold is possibly engaged in binding of nonspecific targets that might have caused the observed growth defect. Together, these findings demonstrate that selection of the scaffold represents a critical step in synthetic sRNA design.

To show the versatile use of our toolset, we simultaneously assembled two synthetic sRNA TUs into pSL0011. The assembly efficiency was reduced to around 10%, which could be caused using six, relatively small, 20 to 104 bp-long DNA fragments. We did not observe additive effects in oxacillin susceptibility by combining the seed regions s8 and s28 targeting the *acrA* transcript (data not shown). However, it shows that the toolset can be utilized to assemble at least two synthetic sRNA TUs. To test the stability of the constructs, we built four different dual synthetic sRNA TUs from maximum to minimum identity (Supporting Information, Figure S9A) and subjected the plasmids to a batch transfer experiment to assess construct stability for 120 h of constant cultivation. All constructs seem to be stable at constant cultivation for at least 48 h; notably, the constructs not sharing the same promoter for both TUs did not show instability over the whole time course of 120 h (Supporting Information, Figure S9B). For the assembly of multiple sRNA TUs, which is beyond the scope of the here presented easy-to-use toolset, we recommend hierarchical Golden Gate assembly strategies such as the modular cloning system^59^ and the construction of dedicated, reusable part libraries.

## Conclusions

Here, we created a set of Golden Gate cloning plasmids for rapid construction and characterization of synthetic sRNAs. The toolset is easily applicable in any molecular biology laboratory in a “plug and play” manner without the need for additional materials. To showcase the system, we rapidly built 37 synthetic RybB variants targeting *acrA* mRNA. Subsequently, the variants were characterized for their ability to render *E. coli* cells sensitive toward the β-lactam antibiotic oxacillin. The workflow of plasmid construction, validation, implementation into the target strain, and acquisition of the growth assay data can be completed in as few as 5 days and is highly scalable.

Seed regions of synthetic sRNAs commonly target the TIR of mRNAs in order to interfere with translation initiation.^16^ Our phenotypic screen of 37 synthetic RybB variants not only confirms that targeting the TIR is an efficient way to increase *acrA*-dependent oxacillin susceptibility but also indicates that the sRNA–mRNA binding energy is an important predictor for the regulatory strength in this particular mRNA region. Since the TIR of mRNAs is often single-stranded to facilitate ribosome binding,^60^ sRNA–mRNA binding energies are hardly influenced by local mRNA secondary structures but mainly depend on the seed sequence and sRNA structure. We therefore recommend the use of tools, such as IntaRNA,^53^ in order to predict the binding energy and, consequently, the regulatory strength of a TIR-targeting sRNA. Even though binding energy predictions and experimental data correlate to a certain extent, our data imply that experimental validation is mandatory to identify the best performing synthetic sRNAs. This is further highlighted by our data for OmrB-s8, which is less efficient to increase oxacillin susceptibility when compared to RybB-s8, MicA-s8, and MicF-s8, probably due to sequestration of the s8 seed region in a stem-loop structure formed with the OmrB scaffold. Consideration of structural features becomes especially important if multiple seed regions are fused upstream of a single scaffold as recently published by Yeom et al. (2022).^61^ Notably, the here promoted toolset would be capable of introducing multiple seed regions upstream of a single scaffold without the need for complex and cumbersome overlap extension PCR procedures. With the respective planning, complex sRNAs can be constructed in a one-pot reaction in a highly parallelized manner.^35^ We show this by constructing dual synthetic TU plasmids in a one-pot reaction, which are stable over a time course of 120 h. Furthermore, we note that our system can be applied to fuse aptamers to sRNAs, as, for example, the MS2 tag for isolation of sRNA-protein complexes.^62^

Our presented sRNA toolkit is an easy-to-use system for post-transcriptional regulation of protein abundances. Synthetic sRNAs can be quickly designed, constructed, and transformed into *E. coli* cells. The system is minimalistic and achieves, in the case of RybB-s8, an approximately threefold reduction of AcrA abundance. In contrast, there is no well-established easy-to-use CRISPR–Cas-based system for post-transcriptional regulation in bacteria. Recently characterized CRISPR–Cas13 and Cas7-11 systems have the potential to serve as tools for post-transcriptional control.^6−10^ However, plasmid-based constructs will be much larger in size based on the proteins to be encoded and will be more complex to ensure controlled co-expression of crRNA and the Cas7-11.

Our work is based on a 16-nt seed region, which is the equivalent size of the natural RybB seed region. Other studies have used longer seed regions, which presumably enhance the translational repression based on their potentially lower binding energies but at the same time increasing the risk of introducing secondary structures.^17,31,61^ Our presented toolset allows the quick alteration not just of the seed region itself but also its properties such as length or the concatenation of multiple seed regions for a multitargeting approach if needed by the user. Taking everything together, we provide a highly versatile and scalable resource for the generation of synthetic sRNAs with a “plug and play” character. Based on our experience with the system, we expect that it will help to speed up the construction and application of synthetic sRNAs in many molecular biology laboratories.

## Materials and Methods

### Culture Conditions, Strains, and Plasmids Used in This Study

*E. coli* K-12 wild-type MG1655 and its derivatives (Table 1) were used for physiological experiments. If not stated otherwise, strains were cultivated in LB medium in Erlenmeyer flasks at 37 °C under continuous shaking at 180 rpm. If appropriate, antibiotics were present in the following concentrations: 100 μg/mL ampicillin (Amp), 50 μg/mL kanamycin (Kan), 15 μg/mL chloramphenicol (Cm), and 6 μg/mL tetracycline (Tet).

**Table 1:** Strains Used in This Study

name	relevant features	reference
*E. coli* MG1655	K-12 F^–^ λ^–^	(63)
*E. coli* DB3.1	F^–^*gyrA462 endA1 glnV44* Δ(*sr1*-*recA*) *mcrB mrr hsdS20*(r_B_^–^, m_B_^–^) *ara14 galK2 lacY1 proA2 rpsL20*(Str^R^) *xyl5* Δ*leu mtl1*	Invitrogen
*E. coli* Top10	F^–^*mcrA* Δ(*mrr*-*hsdRMS*-*mcrBC*) φ80*lacZ*ΔM15 Δ*lacX74 nupG recA1 araD139* Δ(*ara*-*leu*)7697 *galE15 galK16 rpsL*(Str^R^) *endA1* λ^–^	Invitrogen
Δ*acrA*	MG1655 Δ*acrA*::*cat*, Cm^R^	this work
Δ*acrAB*	MG1655 Δ*acrAB*::*cat*, Cm^R^	this work
Δ*rybB*	MG1655 Δ*rybB*::*cat*, Cm^R^	this work
*acrA*–*SD*–*syfp2*	MG1655 *acrA*-*SD*-*syfp2*-*cat*, transcriptional fusion, Cm^R^	this work
*acrA*-9′–*syfp2*	MG1655 *acrA*-*9*′-*syfp2*-*cat*, translational fusion of first 9 *acrA* codons, Cm^R^	this work

**Table 2:** Plasmids Used and Created in This Study (All Plasmid Files are Available as .gbk in the Supporting Information)

name	relevant features	parental plasmid	reference
pSIM5	λ red expression vector, pSC101 *ori*, *repA*^*ts*^, Tet^R^		(64)
pBAD	modified pBAD-TOPO, Amp^R^		(65)
pSL0001	RybB seed region acceptor vector for P_BAD_ expression, Amp^R^	pBAD	this work
pSL0002	seed and scaffold-acceptor vector for P_BAD_ expression, Amp^R^	pSL0001	this work
pSL0003	empty control vector with P_BAD_, Kan^R^	pSL0001	this work
pSL0004	RybB seed region acceptor vector for P_BAD_ expression, Kan^R^	pSL0002	this work
pSL0005	seed and scaffold-acceptor vector for P_BAD_ expression, Kan^R^	pSL0002	this work
pSL0006	empty control vector, Amp^R^	pSL0001	this work
pSL0007	promoter and seed region acceptor vector, Amp^R^	pSL0006	this work
pSL0008	promoter, seed, and scaffold-acceptor vector, Amp^R^	pSL0006	this work
pSL0009	empty control vector, Kan^R^	pSL0003	this work
pSL0010	promoter and seed region acceptor vector, Kan^R^	pSL0009	this work
pSL0011	promoter, seed, and scaffold-acceptor vector, Kan^R^	pSL0009	this work
pSL0137	derivative of pSL0011, SapI recognition sites instead of BbsI, Kan^R^	pSL0011	this work
pSLcol_01	pSL0004 with various seeds (Table S1), Kan^R^	pSL0004	this work
p-P_L_	P_L_lacO-1, Kan^R^	pSL0137	this work
p-P_L_-RybB-s8	RybB-s8 expression from P_L_lacO-1, Kan^R^	pSL0010	this work
p-P_L_-RybB-s38	RybB-s38 expression from P_L_lacO-1, Kan^R^	pSL0010	this work
p-P_L_-RybB-wt	wild-type RybB expression from P_L_lacO-1, Kan^R^	pSL0010	this work
p-P_L_-RybB-Δseed	RybB scaffold expression from P_L_lacO-1, Kan^R^	pSL0010	this work
p-P_L_-MicA-s8	MicA-s8 expression from P_L_lacO-1, Kan^R^	pSL0011	this work
p-P_L_-MicA-Δseed	MicA scaffold expression from P_L_lacO-1, Kan^R^	pSL0011	this work
p-P_L_-MicF-s8	MicF-s8 expression from P_L_lacO-1, Kan^R^	pSL0011	this work
p-P_L_-MicF-Δseed	MicF scaffold expression from P_L_lacO-1, Kan^R^	pSL0011	this work
p-P_L_-OmrB-s8	OmrB-s8 expression from P_L_lacO-1, Kan^R^	pSL0011	this work
p-P_L_-OmrB-Δseed	OmrB scaffold expression from P_L_lacO-1, Kan^R^	pSL0011	this work
p-P_L_-RybB-s28	RybB-s28 expression from P_L_lacO-1, Kan^R^	pSL0010	this work
p-P_L_-RybB-s8-P_L_-RybB-s28	RybB-s8 and RybB-s28 expression from P_L_lacO-1, Kan^R^	pSL0011	this work
p-P_L_-RybB-s8-P_J_-RybB-s28	RybB-s8 and RybB-s28 expression from P_L_lacO-1 and P_BBa_J23119_, respectively, Kan^R^	pSL0011	this work
p-P_L_-RybB-s8-P_L_-MicA-s28	RybB-s8 and MicA-s28 expression from P_L_lacO-1, Kan^R^	pSL0011	this work
p-P_L_-RybB-s8- P_J_-MicA-s28	RybB-s8 and MicA-s28 expression from P_L_lacO-1 and P_BBa_J23119_, respectively, Kan^R^	pSL0011	this work

### Oligodeoxynucleotides

All oligodeoxynucleotides were ordered from Integrated DNA Technologies (IDT, Coralville, USA) or Microsynth Seqlab (Göttingen, Germany) with standard desalting purification (Tables S1 and S2).

### λ Red Recombineering

Gene deletions and chromosomal reporter fusions were constructed using a heat-inducible λ red system as described elsewhere.^64,66,67^ Fragments for λ red recombineering were obtained by PCR and purified using the NucleoSpin gel and PCR clean-up kit (Macherey-Nagel, Düren, Germany). PCR fragments contained 40-bp overhangs on both ends to mediate chromosomal insertion/deletion through homologous recombination. All primers are listed in Table S2. The sequence of the *syfp2*–*cat* template is available as .gbk file in the Supporting Information. For gene deletions, the chloramphenicol acetyltransferase (*cat*) gene was amplified to replace the gene(s) of interest. For the transcriptional *acrA* reporter fusion, the complete *syfp2* open reading frame together with an SD sequence was amplified and inserted immediately behind the *acrA* stop codon. As a result, *acrA* and *syfp2* contained their own SD sequences for independent translation of both genes. For the translational *acrA* reporter fusion, the *syfp2* open reading frame, starting at its second codon, was amplified and fused in frame to the first nine codons of *acrA*. Transcriptional and translational reporter constructs were transcribed from the native *acrA* promoter to retain the *acrA* 5′ UTR. A *cat* gene was present downstream of *syfp2* as a selection marker. For stand-alone genes, a transcriptional terminator sequence was present after the *cat* gene. For genes within operons, the terminator sequence was omitted to enable transcription of downstream genes. All constructs were verified by diagnostic PCR using primers listed in Table S2. After verification, constructs were transduced to a clean *E. coli* MG1655 background using P1 phages according to standard procedures.^68^ Transductants were selected with chloramphenicol and verified by diagnostic PCR as before.

### DNA Assembly

Gibson assembly was used to generate plasmids pSL0001-11 and pSL0137 (cf. Supporting Information for resulting .gbk files) with in-house-generated reaction mix in 10 or 20 μL total volume. All enzymes were supplied by New England Biolabs (NEB, Ipswitch, USA), and reaction mix and procedure were carried out according to Gibson et al. (2009).^41^ Individual parts were amplified using Q5 polymerase. All plasmids containing the *ccdB* gene were transformed into *ccdB* resistant cells (*E. coli* DB3.1), and the Golden Gate cloning sites were verified by either restriction pattern analysis or diagnostic PCR followed by Sanger sequencing (Microsynth SeqLab GmbH, Göttingen, Germany).

For large-scale cloning (plasmid collection pSLcol_01), plasmids were assembled via Golden Gate cloning in 1 μL total reaction volume using an Echo525 (Labcyte, San José, USA). Briefly, annealing of the respective oligonucleotide pairs (Table S1) was performed in annealing buffer (10 mM tris(-HCl) pH 8.0, 50 mM NaCl, and 1 mM EDTA). Reaction mixtures containing 5 fmol of each DNA part (annealed oligonucleotides and plasmid), 0.1 μL of BbsI-HF (NEB, R3539), and 0.1 μL T4 ligase (NEB, M0202) in 1x T4 ligase buffer were generated and incubated in a 384-well PCR cycler (Applied Biosystems, Waltham, USA) with the following program: 20 cycles of 4 min at 16 °C, 3 min at 37 °C followed by 10 min at 50 °C and 10 min at 80 °C, and storage at 4 °C. The Golden Gate assemblies were subsequently transformed into in-house-prepared RbCl competent *E. coli* TOP10 cells.^69^ Briefly, 25 μL of chemically competent cells were added to the reaction mix and incubated for 5–10 min on ice followed by a heat-shock at 42 °C in a 384-well PCR cycler. Cells were then transferred into 96-well deep-well plates (VWR 732–3323) containing 500 μL of LB media and incubated at 37 °C for 30–45 min at 500 rpm on a Multitron HT (Infors, Bottmingen, Switzerland). For selection, 12 transformations at a time were spotted with 20 μL drops onto square agar plates using a multichannel pipette. The plates were tilted (approx. 45° angle) to allow the drops to run down the surface, creating a single line for each transformation, resulting in a cell gradient after overnight cultivation at 37 °C. A representative example of the transformation plate is shown in the Supporting Information (Figure S10). Subsequently, candidates were isolated and picked into 96-well microtiter plates using a colony picking robot (Singer Instruments, Somerset, UK). The resulting candidates were verified via colony PCR using the respective forward seed region primer (Table S1) and one universal reverse primer binding the plasmid (5′-GGT TAT TGT CTC ATG AGC GG-3′). All plasmids in the high-throughput cloning procedure were extracted or purified with open source protocols applying magnetic bead procedures using SeraMag Speed Beads (Cytiva, Marlborough, USA) according to Oberacker et al. (2019).^45^ Strains were cultivated in 1 mL of Sjoerd’s Miniprep Medium (SMM: 16 g/L tryptone, 10 g/L yeast extract, 5 g/L glycerol, and 1x M9 salts) in 96-well deep-well plates with a gas-permeable seal (Thermo Fisher Scientific, AB-0718) at 800 rpm, 37 °C, and 80% relative humidity in an Infors Multitron HT. Transfer of extracted plasmids into new strain backgrounds was performed in 96-well deep-well plates using the TSS transformation procedure according to Chung et al. (1989).^46^ Briefly, fresh colonies (one colony per 3 mL) were picked into an appropriate volume of LB medium (minimum of 200 μL necessary per transformation) and incubated at 37 °C for 1.5 to 2 h. 200 μL of 2x TSS buffer (20% (w/v) PEG 8000, 10% (w/v) DMSO, and 100 nM MgCl_2_ in pH 6.5 LB medium) was aliquoted into 96-well deep-well plates, and 1 μL of the plasmid was added before adding 200 μL of cells. The addition of cells was used to carefully mix the suspension by pipetting up and down. Mixtures were kept on ice for 20–30 min and subsequently incubated in an Infors Multitron HT shaker at 37 °C with 800 rpm for 45–60 min. Twelve TSS transformation mixtures at a time were spotted with 20 μL drops onto agar plates using a multichannel pipette as described above (cf. Supporting Information, Figure S10).

For cloning of constitutive expression plasmids, pSL0010 (containing the RybB scaffold sequence) was used as the acceptor plasmid. Golden Gate cloning using NEB enzymes was applied for assembly of pSL0010 and two short assembly pieces: P_L_lacO-1 promoter and a variable seed region. Oligonucleotide pairs (Table S1) for the short assembly pieces were phosphorylated in 20 μL reaction mixtures containing 100 pmol of each oligonucleotide and 1 μL of T4 polynucleotide kinase (PNK) in 1x T4 DNA ligase buffer. Reaction mixtures were incubated at 37 °C for 1 h, followed by heat inactivation at 65 °C for 20 min. For annealing of phosphorylated oligonucleotide pairs, 2.5 μL of 10x annealing buffer (100 mM tris(-HCl) pH 8.0, 500 mM NaCl, and 10 mM EDTA) and 2.5 μL of dH_2_O were added to the reaction mixtures. The resulting reaction mixtures were incubated for 2 min at 95 °C and cooled down to 25 °C. Finally, assembly reactions were prepared with 100 ng of vector (pSL0010), equimolar amounts of each assembly piece, 1 μL of BbsI-HF (NEB, R3539), and 1 μL of T4 DNA ligase (NEB, M0202) in 1x T4 DNA ligase buffer in a final volume of 15 μL and incubated with the following program: 50 cycles of 4 min at 16 °C, 3 min at 37 °C followed by 10 min at 50 °C and 10 min at 80 °C, and storage at 4 °C. p-P_L_ was constructed by annealing of the two respective oligonucleotides and subsequent Golden Gate assembly into pSL0137 with the only alteration of using SapI instead of BbsI. 5 μL of an assembly reaction was transformed into chemically (CCMB) competent *E. coli* MG1655 cells according to standard procedures.^70^ The resulting expression plasmids were verified by Sanger sequencing (Microsynth Seqlab, Göttingen, Germany).

For Golden Gate cloning with alternative (MicA, MicF, and OmrB) or sRNA TUs, the pSL0011 plasmid was used as the acceptor plasmid. Scaffold fragments were generated by PCR using oligonucleotides containing BbsI recognition sites (Table S1). The P_L_lacO-1, P_BBa_J23119_ promoter, and seed regions were generated by phosphorylation and annealing of oligonucleotides (Table S1) as described above. The reactions contained all DNA fragments in equimolar quantities to the vector. The remaining conditions were the same as described above. The resulting expression plasmids were verified by Sanger sequencing (Microsynth Seqlab, Göttingen, Germany).

### MIC Determination

The MIC was determined according to the broth dilution method. Stationary-phase cultures were diluted 1,000-fold into LB medium, resulting in ∼5 × 10^6^ cfu per mL, and 135 μL was loaded into wells of a transparent 96-well plate (polystyrene, flat bottom; Greiner Bio-One, Kremsmünster, Austria). An oxacillin stock solution (50 mg/mL) and subsequent serial dilutions were prepared in sterile water. 15 μL from each dilution step was added to a defined well to adjust the desired final antibiotic concentration. Plates were sealed with air-permeable polyurethane sealing membranes (Breathe-Easy; Diversified Biotech, Dedham, Massachusetts, USA) and incubated at 37 °C under continuous shaking at 180 rpm for 24 h using a model 3017 orbital shaker (GFL, Burgwedel, Germany). The MIC was defined as the lowest antibiotic concentration that inhibited bacterial growth. As growth control, 15 μL of sterile water without antibiotics was added to one well per strain.

### Phenotypic Screening and Data Analysis

The inducible phenotypic screening in liquid media was performed in an Infinite M Nano^+^ plate reader (Tecan, Männedorf, Switzerland). Briefly, *E. coli* precultures were inoculated in microtiter plates (polystyrene, flat bottom; Greiner Bio-One, Kremsmünster, Austria) overnight at 37 °C, 1000 rpm with 75% relative humidity in 100 μL of LB medium containing kanamycin on an Infors HT incubator (Infors, Bottmingen, Switzerland). Assay cultures were inoculated from the overnight cultures with the Rotor HDA screening robot (Singer Instruments, Somerset, UK) into 96-well microtiter plates containing 200 μL of the respective LB medium supplemented with kanamycin and indicated oxacillin concentrations (0 to 200 μg/mL) with and without the inducing molecule l-arabinose (0.2% final concentration). Plates were sealed with TopSeal-A PLUS (PerkinElmer, Waltham, USA). Measurements were taken with the following program: 120 s orbital shaking (1 mm amplitude) and 120 s linear shaking (1 mm amplitude), followed by wavelength measurement at 600 nm for 24 h. Data were processed with the Growthcurver R package^71^ and visualized with custom R scripts. The AUCs were used to compare the growth.

Solid media screening of constitutively expressed synthetic sRNA constructs was performed based on dilution series of indicated overnight cultures in 96-well flat bottom microtiter plates. A rotor HDA screening robot (Singer Instruments, Somerset, UK) was used to transfer 49 droplets for each dilution into a 7 × 7 grid, using 96-pin pads, onto solid agar plates containing LB, kanamycin, and the indicated oxacillin concentration. Plates were incubated at 37 °C overnight, and images were taken with the PhenoBooth plate documentation system (Singer Instruments, Somerset, UK).

### Measurement of sYFP2 Fluorescence

Chromosomal *syfp2* reporter strains, containing sRNA expression plasmids, were used for sYFP2 measurements. Stationary-phase cultures were diluted 100-fold into LB medium, and 150 μL was loaded into wells of a transparent 96-well plate (polystyrene, flat bottom; Greiner Bio-One, Kremsmünster, Austria). Cells were cultivated in an Infinite M Nano^+^ microplate reader (Tecan, Männedorf, Switzerland) at 37 °C and orbital shaking with an amplitude of 3.5 mm. The optical density was measured at 600 nm (OD_600_), and the sYFP2 fluorescence was monitored using excitation and emission wavelengths of 510 and 540 nm, respectively. The gain was set to 100. Measurements from wells containing pure LB medium were used for background correction of OD_600_ and sYFP2 values. Finally, sYFP2 fluorescence values were normalized to the corresponding OD_600_. Three independent biological replicates were obtained for each strain.

### Northern Blot Analysis

For Northern blot analysis of synthetic sRNAs, expression plasmids were transformed to an *rybB* deletion background. Total RNA was extracted from exponential-phase cultures using the hot acid-phenol method as described.^72^ Northern blot analysis was performed according to a standard protocol.^73^ In short, 10% polyacrylamide gels containing 1x TBE and 7 M urea were used for analysis of sRNAs. After gel separation, RNA was transferred to nylon membranes by semi-dry electroblotting. Oligodeoxynucleotides (Table S2), for detection of specific RNA species, were end-labeled with [γ-^32^P]–ATP using PNK. Prehybridization and hybridization were performed in Church buffer [0.5 M phosphate buffer (pH 7.2), 1% (w/v) bovine serum albumin, 1 mM EDTA, and 7% (w/v) SDS] at 42 °C. After washing of membranes, phosphorimaging was applied for visualization using a Molecular Imager FX (Bio-Rad, Hercules, CA, USA).

### Proteome Analysis

For proteome analysis, cultures (25 mL LB + kanamycin) were grown in quadruplicates at 37 °C in 250 mL baffled flasks on an orbital shaker at 180 rpm (Eppendorf Innova 42). Culture (6 mL) was harvested at an OD_600_ = 0.5 and washed twice in PBS buffer before being stored at −80 °C until sample preparation. Cells were lysed by incubation with 100 μL of a 2% sodium lauroyl sarcosinate (SLS) solution at 95 °C for 15 min and subsequent sonication (VialTweeter; Hielscher, Teltow, Germany). Cell lysates were then reduced by the addition of 5 mM tris(2-carboxyethyl)phosphine and incubation at 95 °C for 15 min, followed by alkylation (10 mM iodoacetamide for 30 min at 25 °C). The cell lysates were cleared by centrifugation, and the total protein was estimated for each sample with a Pierce bicinchoninic acid (BCA) protein assay kit (Thermo Fisher Scientific, Waltham, USA). The cell lysate containing 50 μg of total protein was then diluted with 100 mM ammonium bicarbonate to a final detergent concentration of 0.5% and digested with 1 μg of trypsin (Promega, Madison, Wisconsin, USA) overnight at 30 °C. Next, SLS was removed by precipitation with 1.5% trifluoroacetic acid (TFA) and centrifugation. Peptides were purified using C_18_ microspin columns according to the manufacturer’s instructions (Harvard Apparatus, Holliston, Massachusetts, USA). Purified peptides were dried, resuspended in 50 μL of 0.1% TFA, and analyzed by liquid chromatography–mass spectrometry (MS) carried out on a Exploris 480 instrument connected to an Ultimate 3000 rapid-separation liquid chromatography (RSLC) nano instrument and a nanospray flex ion source (all Thermo Fisher Scientific, Waltham, USA). Peptide separation was performed on a reverse-phase high-performance liquid chromatography (HPLC) column (75 μm by 42 cm) packed in-house with C18 resin (2.4 μm; Dr. Maisch HPLC GmbH, Ammerbuch, Germany). The peptides were first loaded onto a C18 precolumn (preconcentration set-up) and then eluted in the backflush mode with a gradient from 98% solvent A (0.15% formic acid) and 2% solvent B (99.85% acetonitrile and 0.15% formic acid) to 25% solvent B over 65 min, continued from 25 to 35% of solvent B for another 24 min. The flow rate was set to 300 nL/min. The data were acquired in a data-independent mode (DIA) for the initial label-free quantification, and study was set to obtain one high-resolution MS scan at a resolution of 120,000 (*m*/*z* 200) with the scanning range from 320 to 1400 *m*/*z* followed by DIA scans with 27 fixed DIA windows with the width of 24 *m*/*z* (1 *m*/*z* overlap from neighboring windows), ranging from 320 to 970 *m*/*z* at a resolution of 15,000. The automatic gain control was set to 300% for MS survey scans and 3000% for DIA scans. Spectra were identified with the tool DIA-NN^74^ for extracting peptide signals from raw files using a library-free search with an *E. coli* database (UniProt *E.coli* K12 Reference Proteome) (DIA-NN was used with recommended settings). Data analysis and statistic were carried out by the SafeQuant suite^75^ based on the “*report*.*tsv*” of the DIA-NN spectral identification output. Proteins with a protein *q*-value of <0.01 were included for further analysis. DIA-NN outputs were evaluated using a SafeQuant script modified to process DIA-NN outputs, including missing data imputation and statistical evaluation.

### sRNA Binding Affinity Prediction

The interaction energy for the *acrA* mRNA and the sRNA variants was calculated with IntaRNA^53^ version 3.2.0 using the Vienna RNA package 2.4.14 and the “Turner 2004” energy model. IntaRNA was used in the heuristic mode (H) with the seed-extension strategy (model X) with lonely base pairs and GUs at helix ends allowed.

### Statistical Analysis

One-way ANOVA with post-hoc Tukey HSD was applied for pairwise comparison of gene expression data using R statistical language (https://www.r-project.org/). For AUC measurements, Student’s *t*-test (unpaired, two-tailed) was applied.

## Supporting Information Available

Design of seed regions for targeting of *acrA*-mRNA; sRNA secondary structure predictions; phenotypic screening; oxacillin susceptibility assay; secondary structures and seed regions for MicA, MicF, and OmrB; stability assay for assembled constructs; exemplary transformation plate; oligodeoxynucleotides used for Golden Gate cloning; and oligodeoxynucleotides used for λ red recombineering, screening, and Northern blot analysis (PDF)
Genbank sequences for all generated plasmids and the *syfp2*–*cat* template (ZIP)
Mass spectrometry data for the proteins of interest (XLSX)
