# Supplementary material for: An Easy-to-Use Plasmid Toolset for Efficient Generation and Benchmarking of Synthetic Small RNAs in Bacteria
Source: ACS Synth Biol. 2022 Aug 31;11(9):2989–3003. doi: 10.1021/acssynbio.2c00164 (PMC9486967; doi:10.1021/acssynbio.2c00164)
Supplement: Supplementary file 1 — sb2c00164_si_001.pdf [file sb2c00164_si_001.pdf]

Supporting Information

An easy-to-use plasmid toolset for efficient generation and benchmarking of synthetic small RNAs in bacteria

Tania S. Köbel[1,2], Rafael Melo Palhares[1,3], Christin Fromm[3], Witold Szymanski[4], Georgia Angelidou[4], Timo Glatter[4], Jens Georg[5], Bork A. Berghoff[3], Daniel Schindler[1,2]

[1] RG Schindler, Max-Planck-Institute for Terrestrial Microbiology, Karl-von-Frisch-Str. 10, 35043 Marburg, Germany.

[2] MaxGENESYS Biofoundry, Max-Planck-Institute for Terrestrial Microbiology, Karl-von-Frisch-Str. 10, 35043 Marburg, Germany.

[3] Institute for Microbiology and Molecular Biology, Justus Liebig University Giessen, Heinrich-Buff-Ring 26-32, 35392 Giessen, Germany

[4] Mass Spectrometry and Proteomics Core Facility, Max-Planck-Institute for Terrestrial Microbiology, Karl-von-Frisch-Str. 10, 35043 Marburg, Germany.

[5] Albert-Ludwigs-Universität Freiburg, Institut für Biologie III, Schänzlestraße 1, 79104 Freiburg, Germany

This file contains:

Figures S1 to S10

Tables S1 and S2

```
                                                 s5 GTTAAACTTTAGCCTG-5'
                                 s4 TGAAAACTGGTAACTG-5'
                 s3 TCGTTACCCAAATAAT-5'
 s2 TGCTATTATATTTGCG-5'
    ACGATAATATAAACGCAGCAATGGGTTTATTAACTTTTGACCATTGACCAATTTGAAATCGGAC
    +1

                        s30 TTGTCTCCCAAATGCG-5'
                       s29 TTTGTCTCCCAAATGC-5'
                      s28 TTTTGTCTCCCAAATG-5'
                     s27 TTTTTGTCTCCCAAAT-5'
                    s26 GTTTTTGTCTCCCAAA-5'
                   s25 TGTTTTTGTCTCCCAA-5'
                  s24 TTGTTTTTGTCTCCCA-5'
                 s23 CTTGTTTTTGTCTCCC-5'
                s22 ACTTGTTTTTGTCTCC-5'
               s21 TACTTGTTTTTGTCTC-5'
              s20 ATACTTGTTTTTGTCT-5'
             s19 TATACTTGTTTTTGTC-5'
            s18 GTATACTTGTTTTTGT-5'
           s17 TGTATACTTGTTTTTG-5'
          s16 ATGTATACTTGTTTTT-5'
         s15 AATGTATACTTGTTTT-5'
        s14 AAATGTATACTTGTTT-5'
       s13 CAAATGTATACTTGTT-5'
      s12 CCAAATGTATACTTGT-5'
     s11 TCCAAATGTATACTTG-5'
    s10 CTCCAAATGTATACTT-5'
    s9 GCTCCAAATGTATACT-5'
   s8 AGCTCCAAATGTATAC-5'
  s7 GAGCTCCAAATGTATA-5'                                s32 CTACGAGAGTCCGTCG-5'
 s6 TGAGCTCCAAATGTAT-5'                 s31 GAGACCGCCAGCAAGA-5'
    ACTCGAGGTTTACATATGAACAAAAACAGAGGGTTTACGCCTCTGGCGGTCGTTCTGATGCTCTCAGGCAGC

                                                s36 GTCTACGGGCGGCAAC-5'
                                s35 GGTTGTTCCACCGGTC-5'
                s34 CACTGCTGTTTGTCCG-5'
s33 AATCGGGATTGTCCTA-5'
    TTAGCCCTAACAGGATGTGACGACAAACAGGCCCAACAAGGTGGCCAGCAGATGCCCGCCGTTG

                s38 TTGACTTGGAGACGTC-5'
s37 CGCATCATTGTCAGTT-5'
    GCGTAGTAACAGTCAAAACTGAACCTCTGCAGATCACAACCGAGCTTCCGGGTCGCACCAGTGC
    CTACCGGATCGCAGAAGTTCGTCCTCAAGTTAGCGGGATTATCCTGAAGCGTAATTTCAAAGAA
    GGTAGCGACATCGAAGCAGGTGTCTCTCTCTATCAGATTGATCCTGCGACCTATCAGGCGACAT
    ACGACAGTGCGAAAGGTGATCTGGCGAAAGCCCAGGCTGCAGCCAATATCGCGCAATTGACGGT
    GAATCGTTATCAGAAACTGCTCGGTACTCAGTACATCAGTAAGCAAGAGTACGATCAGGCTCTG
    GCTGATGCGCAACAGGCGAATGCTGCGGTAACTGCGGCGAAAGCTGCCGTTGAAACTGCGCGGA
    TCAATCTGGCTTACACCAAAGTCACCTCTCCGATTAGCGGTCGCATTGGTAAGTCGAACGTGAC
    GGAAGGCGCATTGGTACAGAACGGTCAGGCGACTGCGCTGGCAACCGTGCAGCAACTTGATCCG
    ATCTACGTTGATGTGACCCAGTCCAGCAACGACTTCCTGCGCCTGAAACAGGAACTGGCGAATG
    GCACGCTGAAACAAGAGAACGGCAAAGCCAAAGTGTCACTGATCACCAGTGACGGCATTAAGTT
    CCCGCAGGACGGTACGCTGGAATTCTCTGACGTTACCGTTGATCAGACCACTGGGTCTATCACC
    CTACGCGCTATCTTCCCGAACCCGGATCACACTCTGCTGCCGGGTATGTTCGTGCGCGCACGTC
    TGGAAGAAGGGCTTAATCCAAACGCTATTTTAGTCCCGCAACAGGGCGTAACCCGTACGCCGCG
    TGGCGATGCCACCGTACTGGTAGTTGGCGCGGATGACAAAGTGGAAACCCGTCCGATCGTTGCA
    AGCCAGGCTATTGGCGATAAGTGGCTGGTGACAGAAGGTCTGAAAGCAGGCGATCGCGTAGTAA
    TAAGTGGGCTGCAGAAAGTGCGTCCTGGTGTCCAGGTAAAAGCACAAGAAGTTACCGCTGATAA
    TAACCAGCAAGCCGCAAGCGGTGCTCAGCCTGAACAGTCCAAGTCTTAA
```

Figure S1. Design of seed regions for targeting of *acrA*. The *acrA* sequence is given in black letters. Green and red letters indicate start and stop codons, respectively. Orange letters indicate the 'five codon window'. The Shine-Dalgarno sequence is highlighted in a grey box. +1 indicates the transcriptional start site. Seed regions for *acrA* binding are given in blue letters. To illustrate complementarity to *acrA*, the 5' end of seed regions is on the right-hand side. Seed regions are complementary to the 5' UTR (s2-s5), the TIR (s6-s13), the start codon (s14-s23), the 'five codon window' (s24-s30) and the coding region (s31-s38).

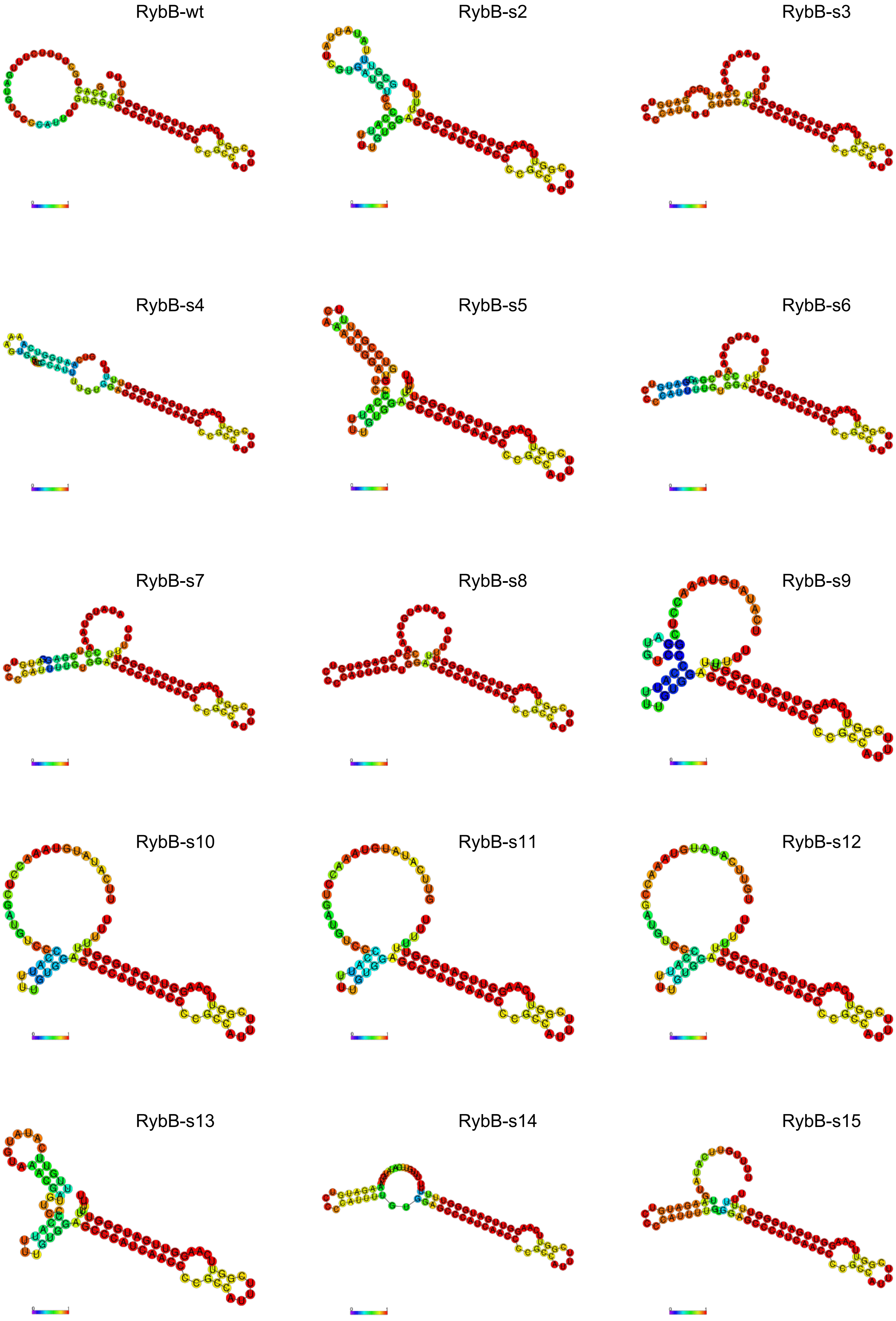

RybB-wt
RybB-s2
RybB-s3
RybB-s4
RybB-s5
RybB-s6
RybB-s7
RybB-s8
RybB-s9
RybB-s10
RybB-s11
RybB-s12
RybB-s13
RybB-s14
RybB-s15

S3

RybB-16

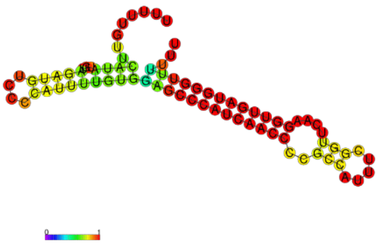

RybB-s17

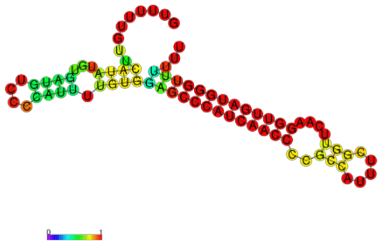

RybB-s18

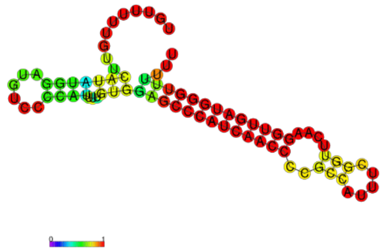

RybB-s19

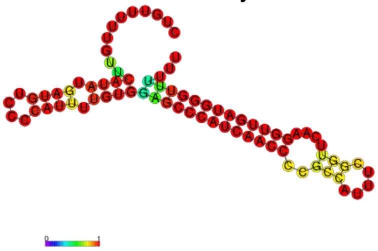

RybB-s20

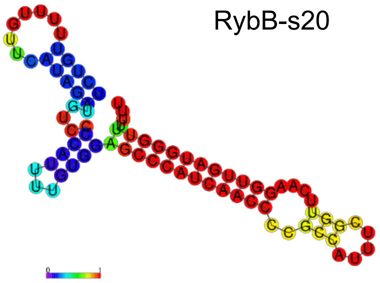

RybB-s21

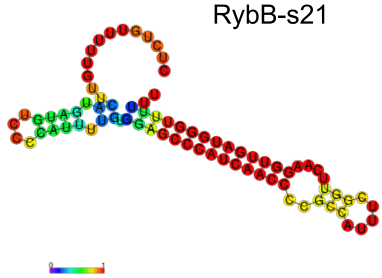

RybB-s22

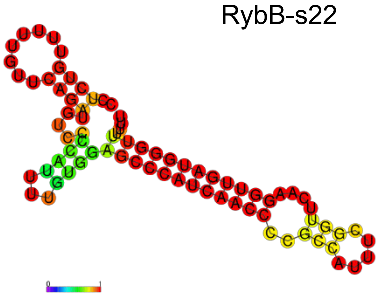

RybB-s23

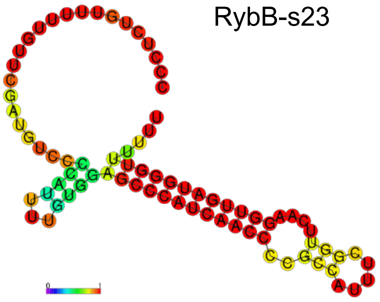

RybB-s24

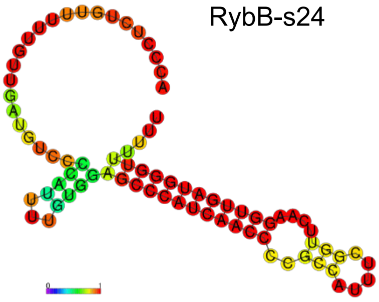

RybB-s25

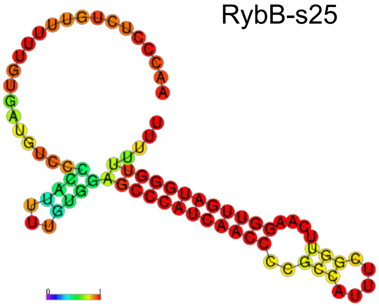

RybB-s26

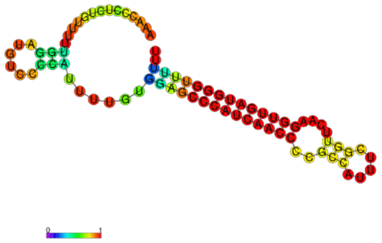

RybB-s27

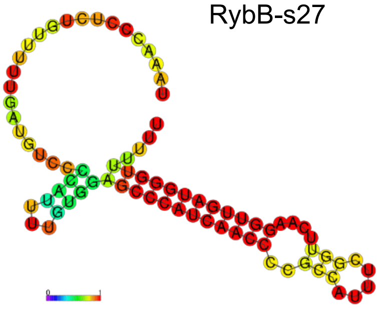

RybB-s28

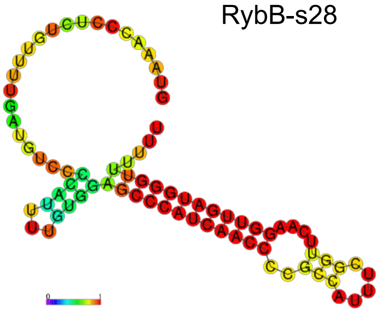

RybB-s29

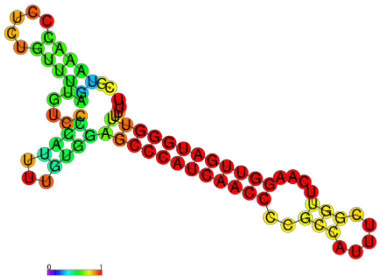

RybB-s30

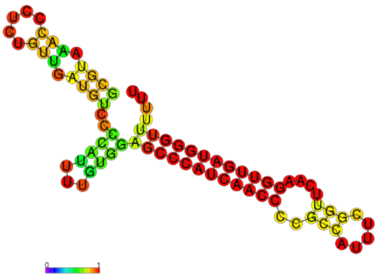

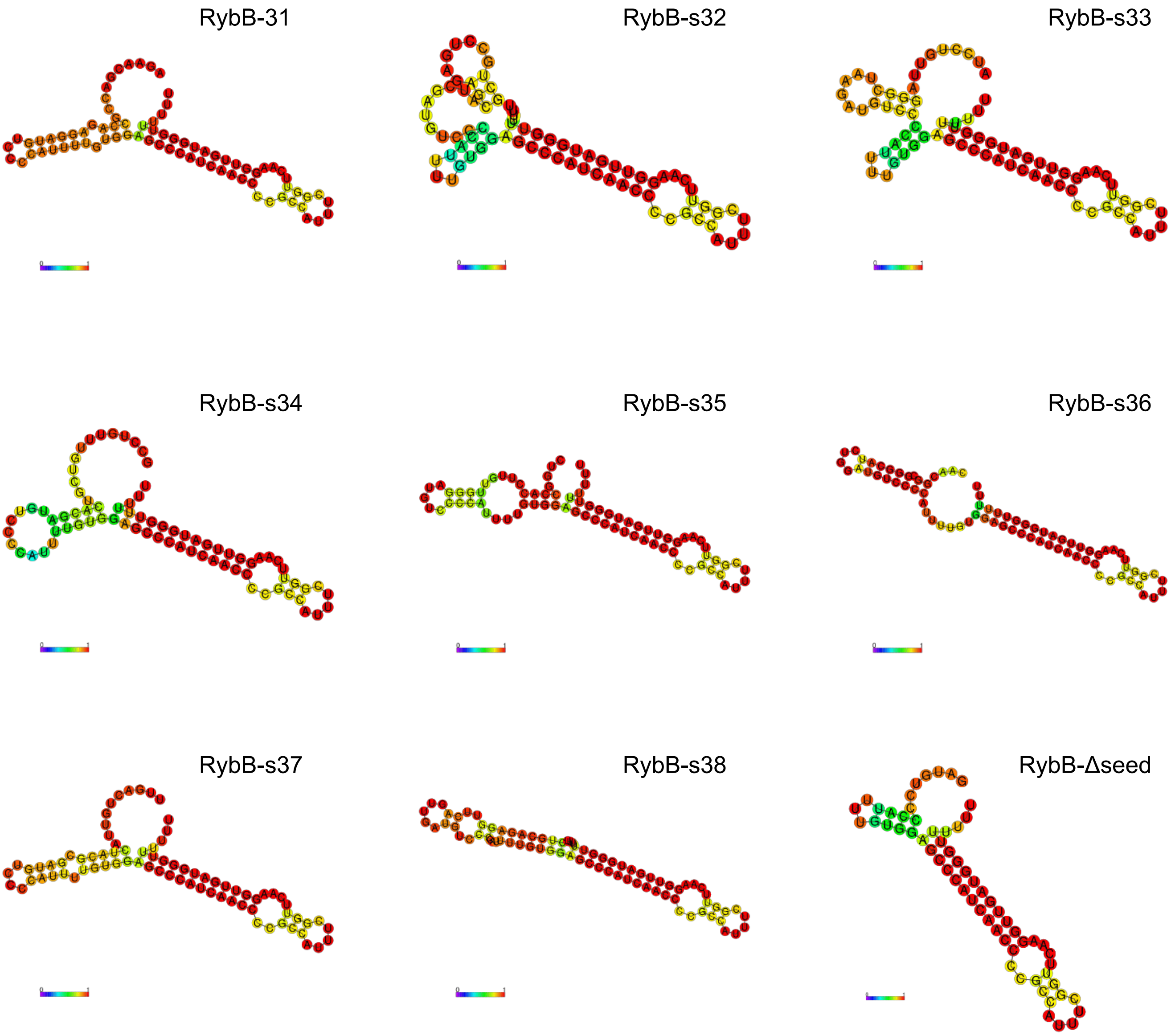

Figure S2. Secondary structure predictions for synthetic RybB sRNAs. Secondary structures were predicted using the RNAfold web server (http://rna.tbi.univie.ac.at/cgi-bin/RNAWebSuite/RNAfold.cgi). The colored bar indicates the base-pair probabilities. Wild-type (wt) RybB and the RybB scaffold (RybB-Δseed) are shown for comparison.

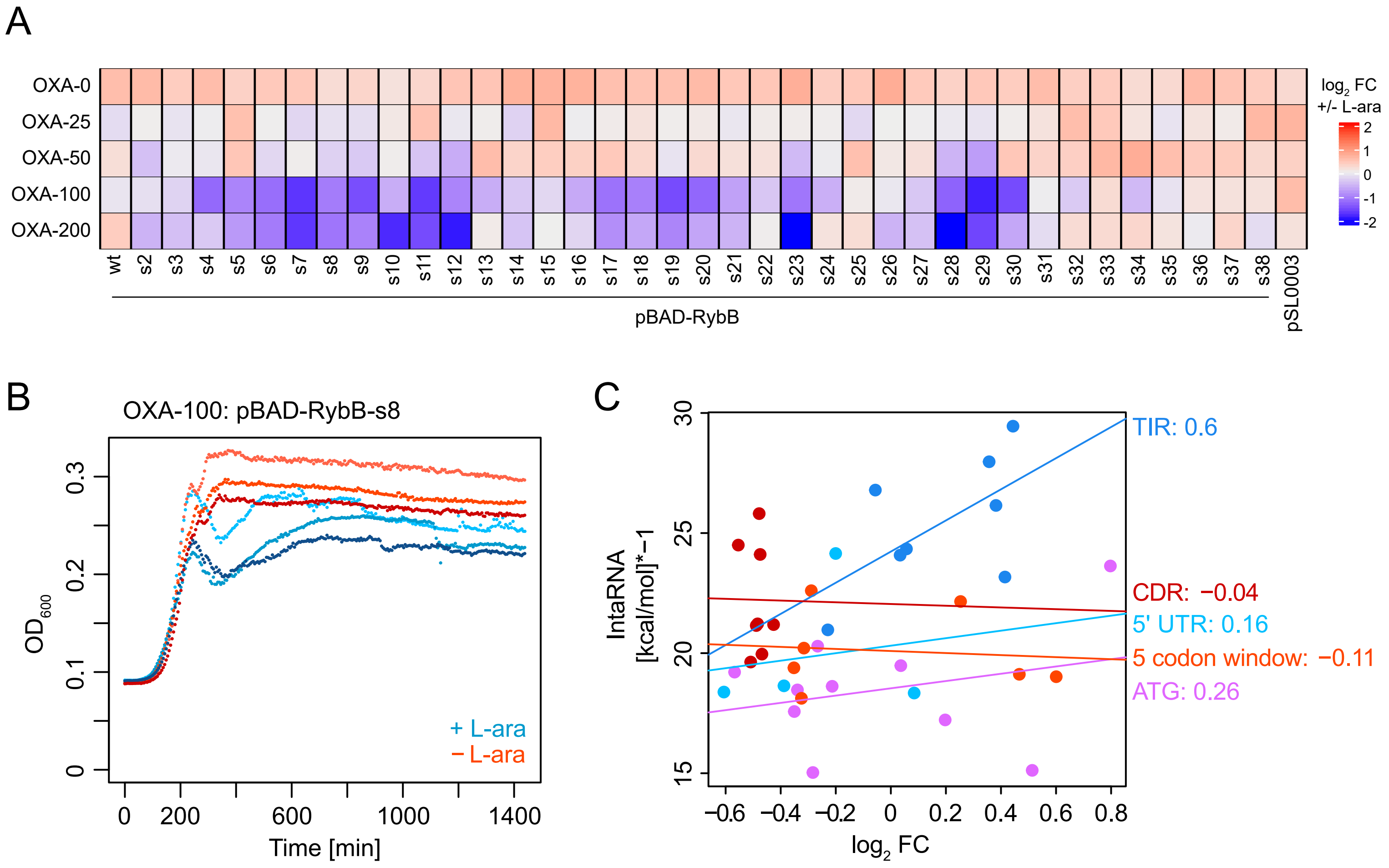

Figure S3. Phenotypic screening of synthetic RybB sRNAs with seed regions s2-s38. Seed regions were cloned into the pBAD derivative pSL0004 for inducible expression of synthetic RybB sRNAs. Stationary-phase cultures were inoculated in 96-well plates to monitor growth (OD_{600}) in a plate reader. **(A)** LB medium contained oxacillin (OXA) at the indicated concentrations (0-200 µg/ml). Strains were treated with L-arabinose (+ L-ara) to induce sRNA expression or left untreated (- L-ara). The areas under the curves (AUC) were calculated and AUC values were subsequently used to calculate log_2 fold-changes (FC). Log_2 FC are illustrated in a heatmap. Wild-type (wt) RybB and the empty plasmid pSL0003 served as controls. **(B)** Growth curves for pBAD-RybB-s8 at 100 µg/ml oxacillin (OXA-100) with (blue) and without (red) addition of L-arabinose (L-ara). The dots show the measured optical density (OD) at 600 nm. The presence of oxacillin causes irregular growth curves with peaks at ~300 min due to cellular filamentation. **(C)** Correlation analysis of log_2 FC (-/+ L-ara) and IntaRNA energy predictions for sRNA-*acrA* pairs (multiplied by -1). Numbers at the right-hand side represent Pearson's *r* for individual subsets (5' UTR: 5' untranslated region; TIR: translation initiation region; ATG: start codon; CDR: coding region).

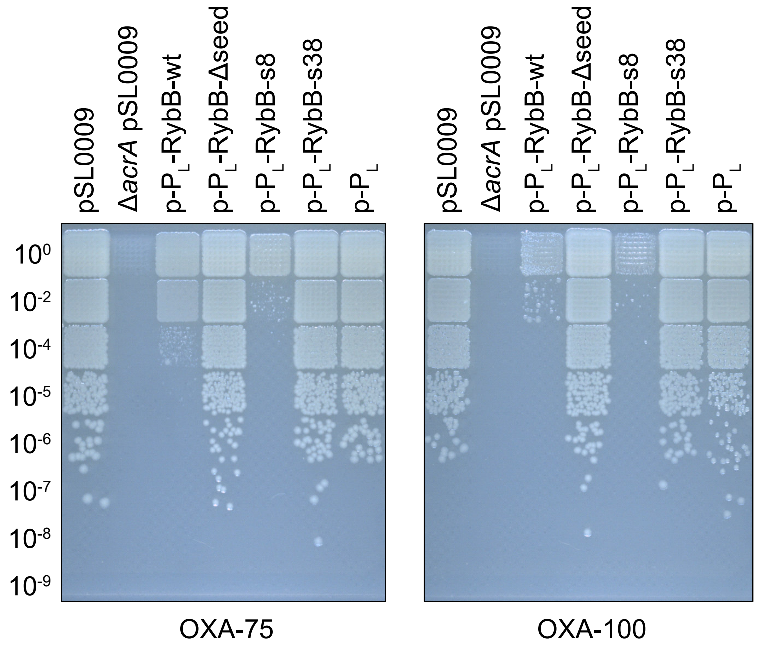

Figure S4. Oxacillin susceptibility assay. Stationary-phase cultures were serially diluted as indicated and spotted onto LB agar plates containing varying concentrations of oxacillin (OXA, 75 and 100 µg/ml; *cf*. Fig. 3E for 0-50 µg/ml). Plates were incubated overnight at 37 °C. The empty plasmid pSL0009, wild-type RybB (RybB-wt), RybB lacking a seed region (RybB-Δseed) and a plasmid containing the $P_L$lacO-1 promoter (p-P_L) were used as controls.

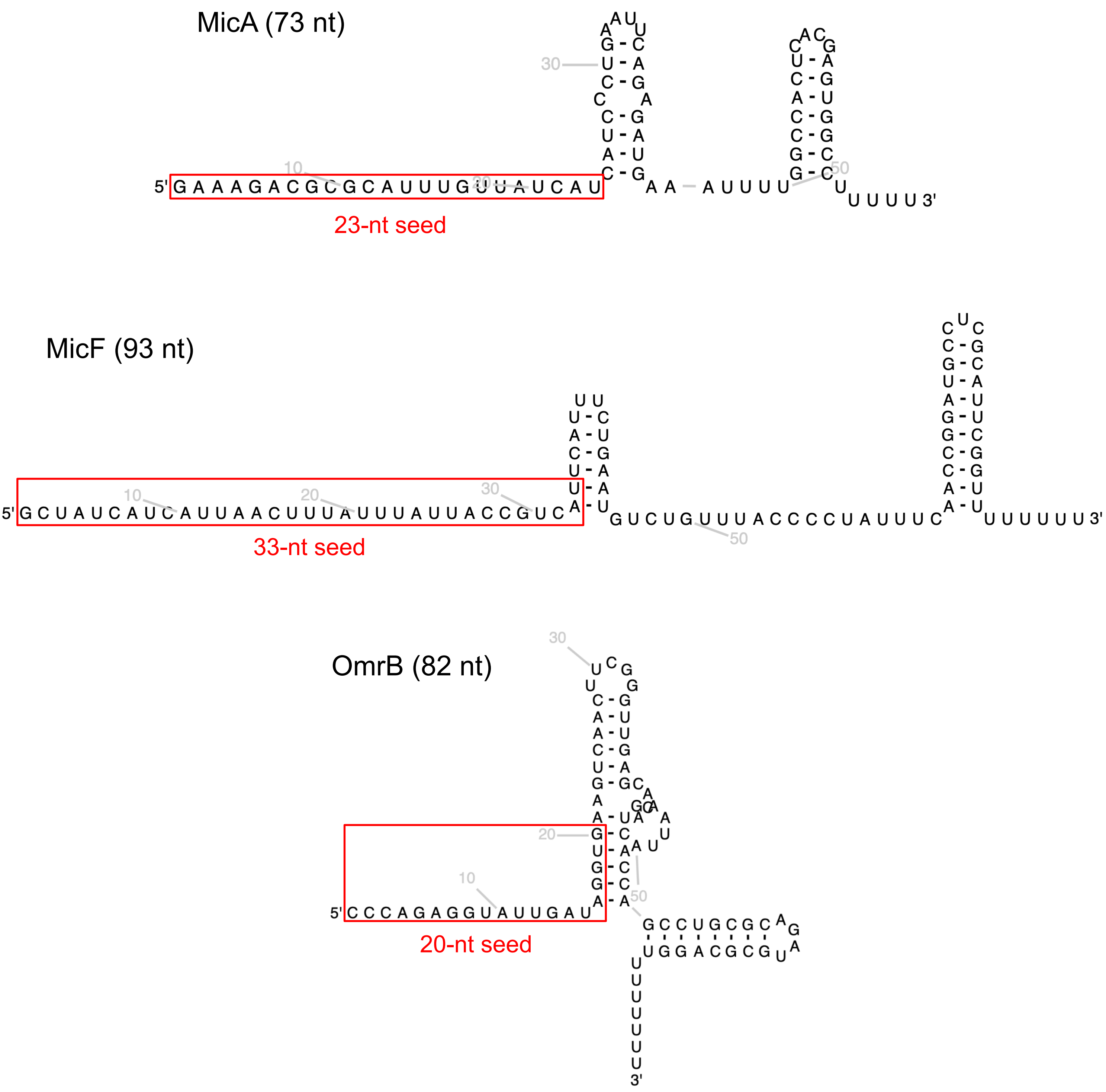

Figure S5. Secondary structures and seed regions for MicA, MicF and OmrB. Secondary structures were retrieved from RNAcentral (https://rnacentral.org/). Corresponding seed regions are marked in red.

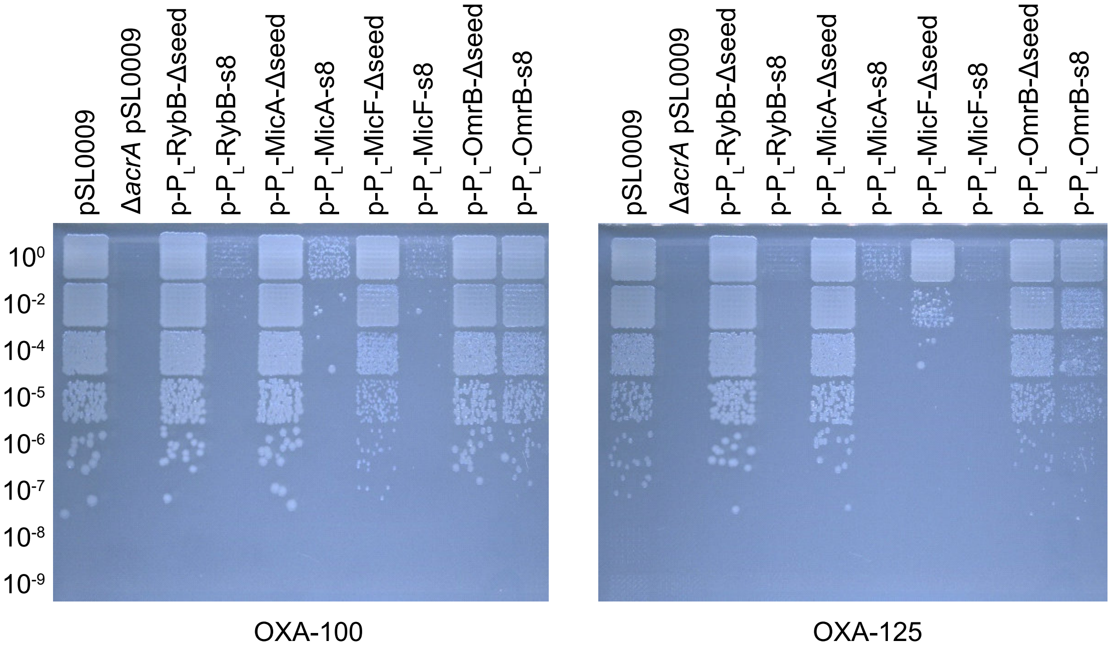

Figure S6. Oxacillin susceptibility assay. Stationary-phase cultures were serially diluted as indicated and spotted onto LB agar plates containing varying concentrations of oxacillin (OXA, 100 and 125 µg/ml; *cf*. Fig. 5 for 0-75 µg/ml). Plates were incubated overnight at 37 °C. Synthetic sRNAs containing the s8 seed region were compared to variants lacking a seed region (Δseed). The empty plasmid pSL0009 was used as a control.

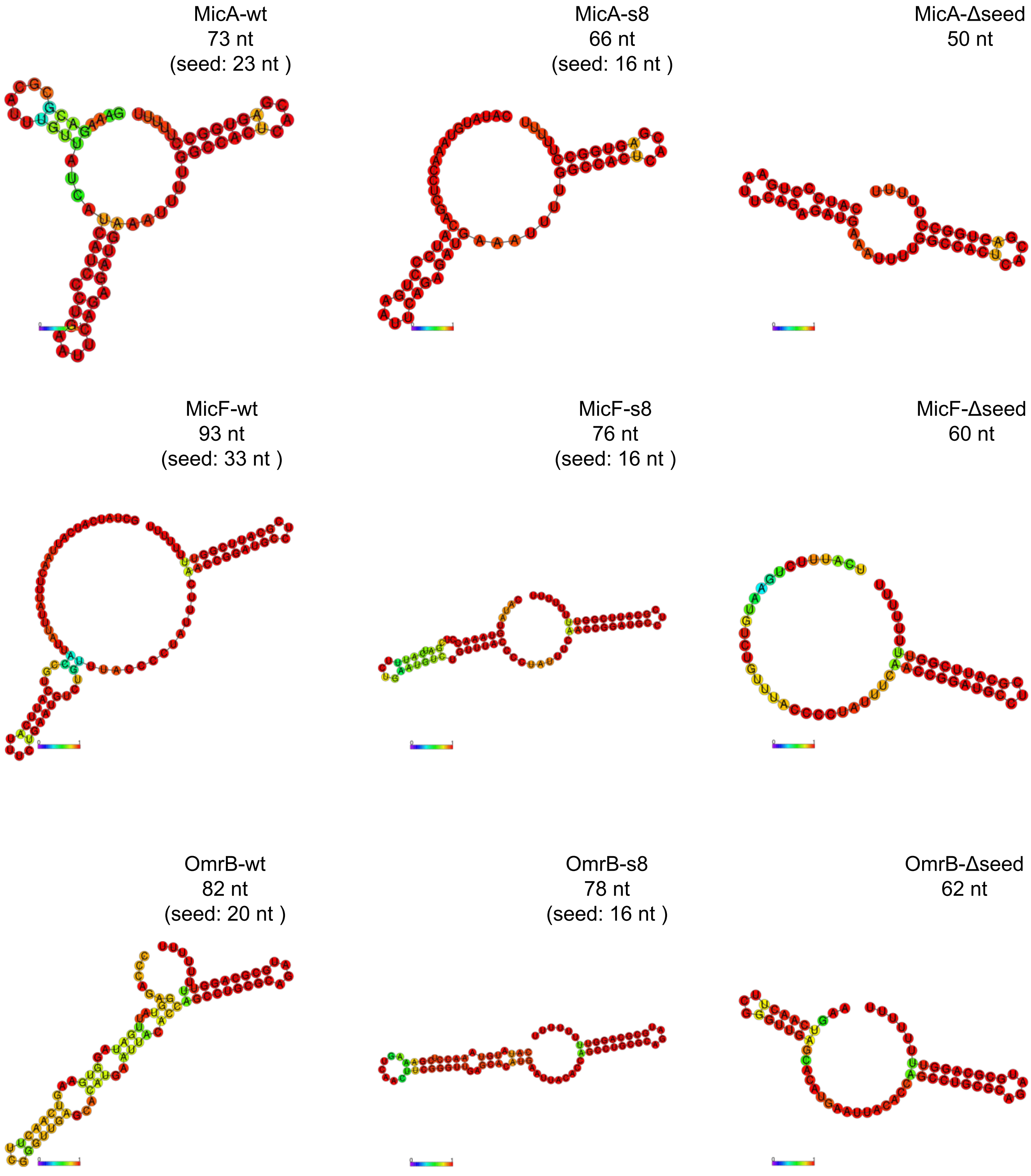

Figure S7. Secondary structure predictions for synthetic sRNAs containing seed region s8. Secondary structures were predicted using the RNAfold web server (http://rna.tbi.univie.ac.at/cgi-bin/RNAWebSuite/RNAfold.cgi). The colored bar indicates the base-pair probabilities. Wild-type (wt) MicA, MicF and OmrB sRNAs and variants lacking a seed region (Δseed) are shown for comparison.

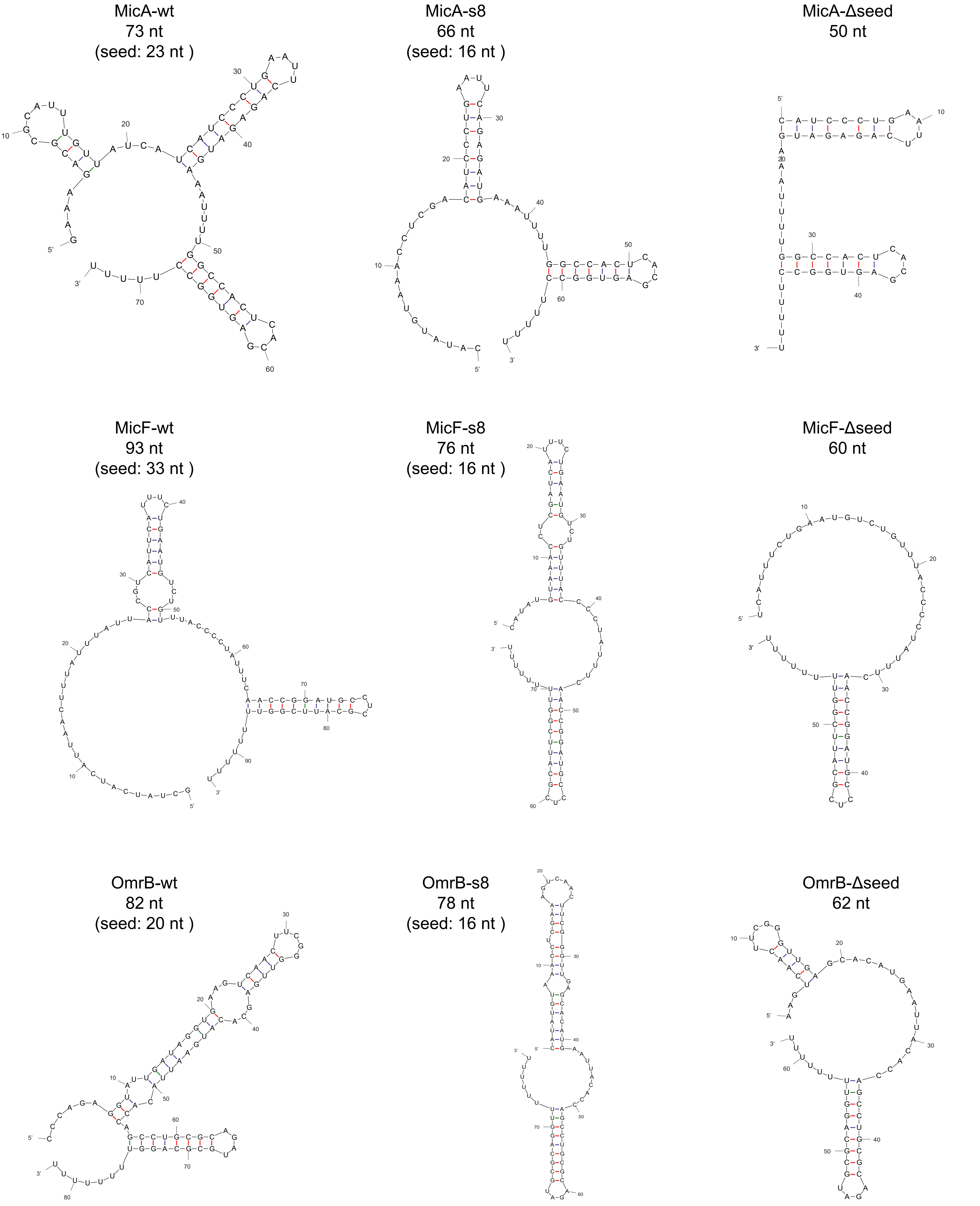

Figure S8. Secondary structure predictions for synthetic sRNAs containing seed region s8. Secondary structures were predicted using the Mfold web server (http://www.unafold.org/mfold/applications/rna-folding-form.php). Wild-type (wt) MicA, MicF and OmrB sRNAs and variants lacking a seed region (Δseed) are shown for comparison.

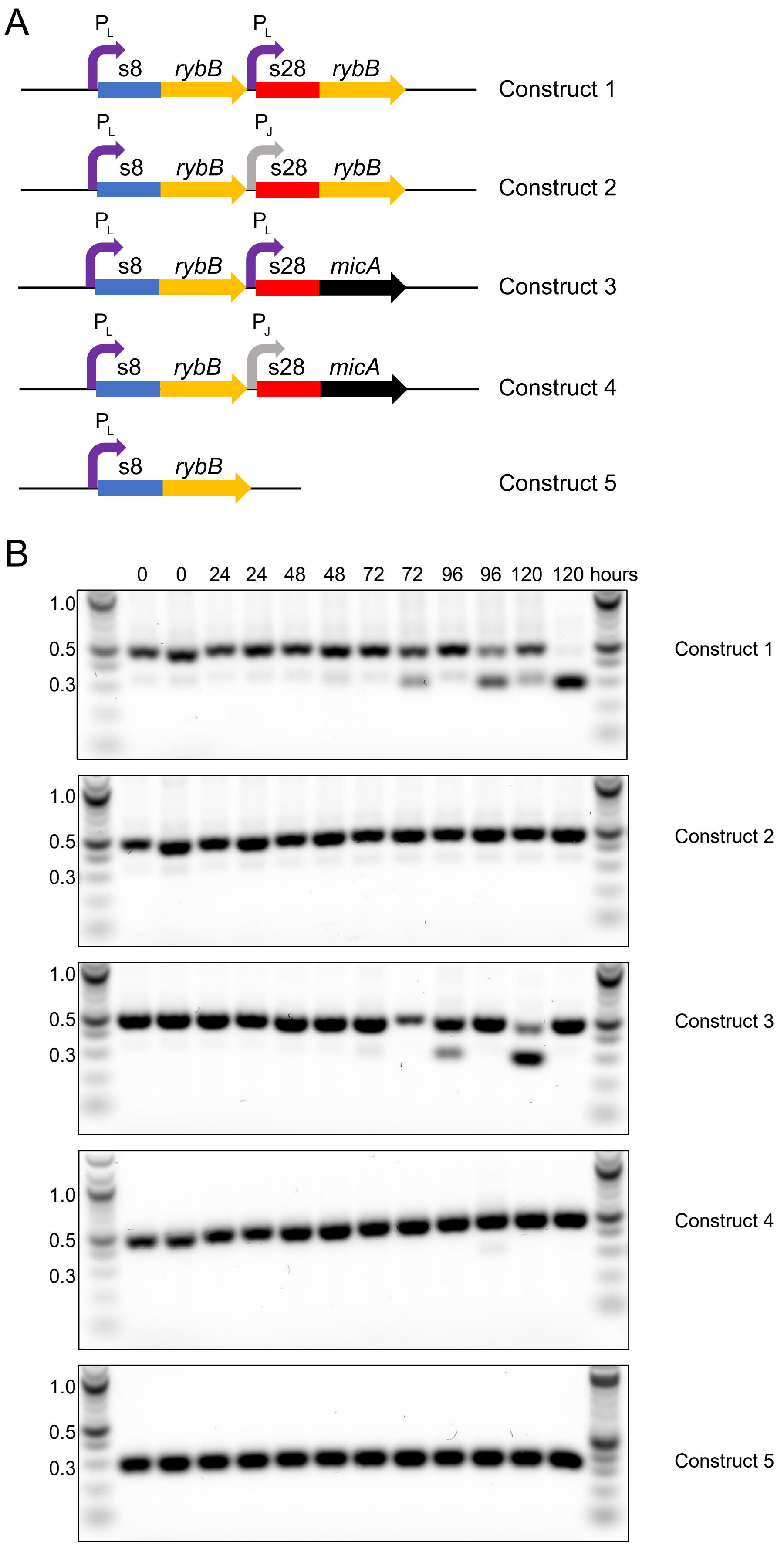

Figure S9. Stability assay for assembled constructs. **(A)** Constructs with dual sRNA TUs were assembled using high-complexity Golden Gate cloning with plasmid pSL0011 (Constructs 1-4; ranging from high identity to no identity). A construct with a single sRNA TU was used for comparison (Construct 5). Colors indicate identity of DNA parts. **(B)** Clones containing constructs were incubated separately (as biological duplicates) in a 96-well flat-bottom plate with 150 µl of LB and 50 µg/ml kanamycin per well. The plate was incubated at 180 rpm and 37 °C. After a 24-hours period, 1 µl of the cell suspension was collected in 50 µl dH_2O and frozen for a screening PCR. At the same time, 1 µl of the cell suspension was inoculated in a new plate containing 150 µl LB and 50 µg/ml kanamycin for a new incubation period. This was repeated for five days (120 hours). The screening PCR was performed in a total volume of 10 µl, consisting of 5 µl OneTaq Quick-Load 2x Master Mix (NEB), 1 µl of frozen cell samples, 2 µl dH_2O, and 1 µl of each 10 µM colony PCR primer (Table S2). The resulting PCR fragments were separated in a 2% agarose gel.

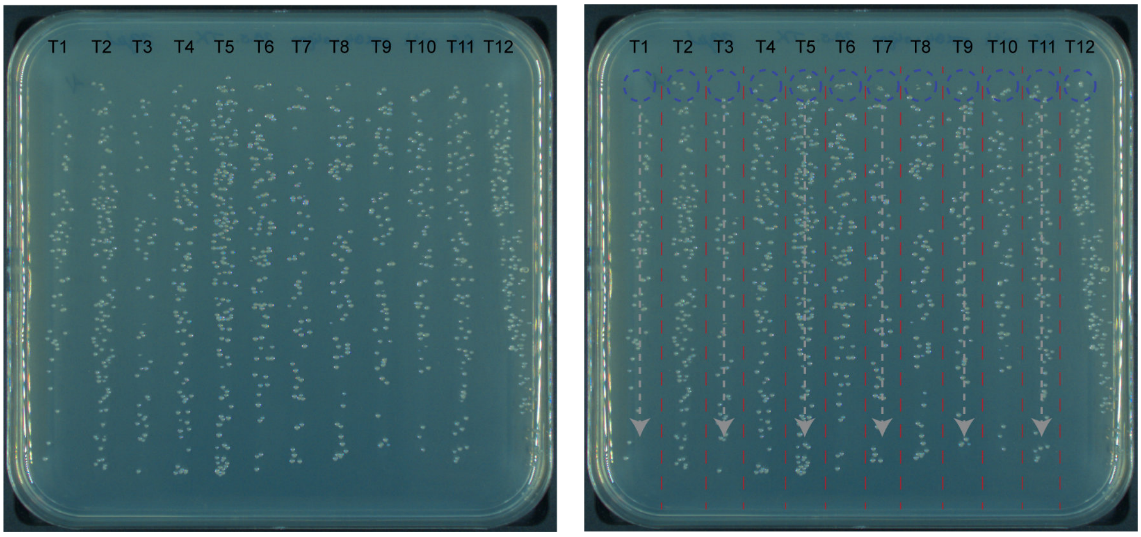

Figure S10. Exemplary transformation plate of the high-throughput plating procedure. Twelve transformation mixtures were plated in parallel by gravity flow of 20 µl drops using a multi-channel pipette. Both panels show the same representative plate. The left panel shows the transformation plate without explanatory features, just the indication of the individual transformation (T1 to T12). The right panel indicates the positions where the drops were set (blue dotted circles). Grey arrows indicate the gravity flow direction. Red dotted lines separate the individual transformations (lanes) for clarification.

Oligodeoxynucleotides

All oligodeoxynucleotides were ordered from Integrated DNA Technologies (IDT, Coralville, USA) or Microsynth Seqlab (Göttingen, Germany) with standard desalting purification.

Table S1. Oligodeoxynucleotides used for Golden Gate cloning.

Forward sequence [5'-3']	Reverse sequence [5'-3']	Assembly piece	Resulting plasmid
**CCAT**GCCACTGCTTTTCTTT	**CATC**AAAGAAAAGCAGTGGC	16 nt native RybB seed region, variant 1	pSLcol_01.s01<br>p-P_L-RybB-wt
**CCAT**GCGTTTATATTATCGT	**CATC**ACGATAATATAAACGC	16 nt seed region, variant 2, target: *acrA*	pSLcol_01.s02
**CCAT**TAATAAACCCATTGCT	**CATC**AGCAATGGGTTTATTA	16 nt seed region, variant 3, target: *acrA*	pSLcol_01.s03
**CCAT**GTCAATGGTCAAAAGT	**CATC**ACTTTTGACCATTGAC	16 nt seed region, variant 4, target: *acrA*	pSLcol_01.s04
**CCAT**GTCCGATTTCAAATTG	**CATC**CAATTTGAAATCGGAC	16 nt seed region, variant 5, target: *acrA*	pSLcol_01.s05
**CCAT**TATGTAAACCTCGAGT	**CATC**ACTCGAGGTTTACATA	16 nt seed region, variant 6, target: *acrA*	pSLcol_01.s06
**CCAT**ATATGTAAACCTCGAG	**CATC**CTCGAGGTTTACATAT	16 nt seed region, variant 7, target: *acrA*	pSLcol_01.s07
**CCAT**CATATGTAAACCTCGA	**CATC**TCGAGGTTTACATATG	16 nt seed region, variant 8, target: *acrA*	pSLcol_01.s08<br>p-P_L-RybB-s8
	**GATG**TCGAGGTTTACATATG	16 nt seed region, variant 8, target: *acrA*	p-P_L-MicA-s8
	**ATGA**TCGAGGTTTACATATG	16 nt seed region, variant 8, target: *acrA*	p-P_L-MicF-s8
	**ACTT**TCGAGGTTTACATATG	16 nt seed region, variant 8, target: *acrA*	p-P_L-OmrB-s8
**CCAT**TCATATGTAAACCTCG	**CATC**CGAGGTTTACATATGA	16 nt seed region, variant 9, target: *acrA*	pSLcol_01.s09
**CCAT**TTCATATGTAAACCTC	**CATC**GAGGTTTACATATGAA	16 nt seed region, variant 10, target: *acrA*	pSLcol_01.s10
**CCAT**GTTCATATGTAAACCT	**CATC**AGGTTTACATATGAAC	16 nt seed region, variant 11, target: *acrA*	pSLcol_01.s11
**CCAT**TGTTCATATGTAAACC	**CATC**GGTTTACATATGAACA	16 nt seed region, variant 12, target: *acrA*	pSLcol_01.s12
**CCAT**TTGTTCATATGTAAAC	**CATC**GTTTACATATGAACAA	16 nt seed region, variant 13, target: *acrA*	pSLcol_01.s13
**CCAT**TTTGTTCATATGTAAA	**CATC**TTTACATATGAACAAA	16 nt seed region, variant 14, target: *acrA*	pSLcol_01.s14
**CCAT**TTTTGTTCATATGTAA	**CATC**TTACATATGAACAAAA	16 nt seed region, variant 15, target: *acrA*	pSLcol_01.s15
**CCAT**TTTTTGTTCATATGTA	**CATC**TACATATGAACAAAAA	16 nt seed region, variant 16, target: *acrA*	pSLcol_01.s16
**CCAT**GTTTTTGTTCATATGT	**CATC**ACATATGAACAAAAAC	16 nt seed region, variant 17, target: *acrA*	pSLcol_01.s17
**CCAT**TGTTTTTGTTCATATG	**CATC**CATATGAACAAAAACA	16 nt seed region, variant 18, target: *acrA*	pSLcol_01.s18
**CCAT**CTGTTTTTGTTCATAT	**CATC**ATATGAACAAAAACAG	16 nt seed region, variant 19, target: *acrA*	pSLcol_01.s19
**CCAT**TCTGTTTTTGTTCATA	**CATC**TATGAACAAAAACAGA	16 nt seed region, variant 20, target: *acrA*	pSLcol_01.s20
**CCAT**CTCTGTTTTTGTTCAT	**CATC**ATGAACAAAAACAGAG	16 nt seed region, variant 21, target: *acrA*	pSLcol_01.s21
**CCAT**CCTCTGTTTTTGTTCA	**CATC**TGAACAAAAACAGAGG	16 nt seed region, variant 22, target: *acrA*	pSLcol_01.s22
**CCAT**CCCTCTGTTTTTGTTC	**CATC**GAACAAAAACAGAGGG	16 nt seed region, variant 23, target: *acrA*	pSLcol_01.s23
**CCAT**ACCCTCTGTTTTTGTT	**CATC**AACAAAAACAGAGGGT	16 nt seed region, variant 24, target: *acrA*	pSLcol_01.s24
**CCAT**AACCCTCTGTTTTTGT	**CATC**ACAAAAACAGAGGGTT	16 nt seed region, variant 25, target: *acrA*	pSLcol_01.s25
**CCAT**AAACCCTCTGTTTTTG	**CATC**CAAAAACAGAGGGTTT	16 nt seed region, variant 26, target: *acrA*	pSLcol_01.s26
**CCAT**TAAACCCTCTGTTTTT	**CATC**AAAAACAGAGGGTTTA	16 nt seed region, variant 27, target: *acrA*	pSLcol_01.s27
**CCAT**GTAAACCCTCTGTTTT	**CATC**AAAACAGAGGGTTTAC	16 nt seed region, variant 28, target: *acrA*	pSLcol_01.s28
**CCAT**CGTAAACCCTCTGTTT	**CATC**AAACAGAGGGTTTACG	16 nt seed region, variant 29, target: *acrA*	pSLcol_01.s29
**CCAT**GCGTAAACCCTCTGTT	**CATC**AACAGAGGGTTTACGC	16 nt seed region, variant 30, target: *acrA*	pSLcol_01.s30
**CCAT**AGAACGACCGCCAGAG	**CATC**CTCTGGCGGTCGTTCT	16 nt seed region, variant 31, target: *acrA*	pSLcol_01.s31
**CCAT**GCTGCCTGAGAGCATC	**CATC**GATGCTCTCAGGCAGC	16 nt seed region, variant 32, target: *acrA*	pSLcol_01.s32
**CCAT**ATCCTGTTAGGGCTAA	**CATC**TTAGCCCTAACAGGAT	16 nt seed region, variant 33, target: *acrA*	pSLcol_01.s33
**CCAT**GCCTGTTTGTCGTCAC	**CATC**GTGACGACAAACAGGC	16 nt seed region, variant 34, target: *acrA*	pSLcol_01.s34
**CCAT**CTGGCCACCTTGTTGG	**CATC**CCAACAAGGTGGCCAG	16 nt seed region, variant 35, target: *acrA*	pSLcol_01.s35
**CCAT**CAACGGCGGGCATCTG	**CATC**CAGATGCCCGCCGTTG	16 nt seed region, variant 36, target: *acrA*	pSLcol_01.s36
**CCAT**TTGACTGTTACTACGC	**CATC**GCGTAGTAACAGTCAA	16 nt seed region, variant 37, target: *acrA*	pSLcol_01.s37
**CCAT**CTGCAGAGGTTCAGTT	**CATC**AACTGAACCTCTGCAG	16 nt seed region, variant 38 for *acrA*	pSLcol_01.s38<br>p-P_L-RybB-s38
**ACCA**TTGTGAGCGGATAACAA<br>TTGACATTGTGAGCGGATAAC<br>AAGATACTGAGCAC	**ATGG**GTGCTCAGTATCTTGTT<br>ATCCGCTCACAATGTCAATTG<br>TTATCCGCTCACAA	$P_L$lacO-1 promoter	p-P_L-RybB-wt<br>p-P_L-RybB-s8<br>p-P_L-RybB-s28<br>p-P_L-RybB-s38<br>p-P_L-MicA-s8<br>p-P_L-MicF-s8<br>p-P_L-OmrB-s8<br>p-P_L-s8-RybB-P_L-s28-RybB<br>p-P_L-s8-RybB-P_J-s28-RybB<br>p-P_L-s8-RybB-P_L-s28-MicA<br>p-P_L-s8-RybB-P_J-s28-MicA
	**CATC**GTGCTCAGTATCTTGTT<br>ATCCGCTCACAATGTCAATTG<br>TTATCCGCTCACAA	$P_L$lacO-1 promoter	p-P_L-RybB-Δseed

	**GATG**GTGCTCAGTATCTTGTT<br>ATCCGCTCACAATGTCAATTG<br>TTATCCGCTCACAA	$P_L$lacO-1 promoter	p-P_L-MicA-Δseed
	**ATGA**GTGCTCAGTATCTTGTT<br>ATCCGCTCACAATGTCAATTG<br>TTATCCGCTCACAA	$P_L$lacO-1 promoter	p-P_L-MicF-Δseed
	**ACTT**GTGCTCAGTATCTTGTT<br>ATCCGCTCACAATGTCAATTG<br>TTATCCGCTCACAA	$P_L$lacO-1 promoter	p-P_L-OmrB-Δseed
**TTC**TTGTGAGCGGATAACAAT<br>TGACATTGTGAGCGGATAACA<br>AGATACTGAGCACCCATG	**CCG**CATGGGTGCTCAGTATCT<br>TGTTATCCGCTCACAATGTCA<br>ATTGTTATCCGCTCACAA	$P_L$lacO-1 promoter	p-P_L
GCGAAGACAA**CATC**CCTGAAT<br>TCAGAGATG	GCGAAGACAA**ATCC**GATACCG<br>AACCGTTTGCG	MicA scaffold	p-P_L-MicA-s8<br>p-P_L-MicA-Δseed
GCGAAGACAA**TCAT**TTCTGAA<br>TGTCTGTTTAC	GCGAAGACAA**ATCC**CTGTGGT<br>AGCACAGAATAATG	MicF scaffold	p-P_L-MicF-s8<br>p-P_L-MicF-Δseed
GCGAAGACAA**AAGT**CAACTTC<br>GGGTTGAG	GCGAAGACAA**ATCC**GTCGGTT<br>ACTGTTACAGATTG	OmrB scaffold	p-P_L-OmrB-s8<br>p-P_L-OmrB-Δseed
**CCAT**CATATGTAAACCTCGA	**GTAC**TCGAGGTTTACATATG	16 nt seed region, variant 8, target: *acrA*	p-P_L-s8-RybB-P_L-s28-RybB<br>p-P_L-s8-RybB-P_J-s28-RybB<br>p-P_L-s8-RybB-P_L-s28-MicA<br>p-P_L-s8-RybB-P_J-s28-MicA
**CTTG**GTAAACCCTCTGTTTT	**TGCA**AAAACAGAGGGTTTAC	16 nt seed region, variant 28, target: *acrA*	p-P_L-s8-RybB-P_L-s28-RybB<br>p-P_L-s8-RybB-P_J-s28-RybB<br>p-P_L-s8-RybB-P_L-s28-MicA<br>p-P_L-s8-RybB-P_J-s28-MicA
**GCAA**TTGTGAGCGGATAACAA<br>TTGACATTGTGAGCGGATAAC<br>AAGATACTGAGCAC	**CAAG**GTGCTCAGTATCTTGTT<br>ATCCGCTCACAATGTCAATTG<br>TTATCCGCTCACAA	$P_L$lacO-1 promoter	p-P_L-s8-RybB-P_L-s28-RybB<br>p-P_L-s8-RybB-P_L-s28-MicA
**GCAA**TTGACAGCTAGCTCAGT<br>CCTAGGTATAATGCTAGC	**CAAG**GCTAGCATTATACCTAG<br>GACTGAGCTAGCTGTCAA	P_{BBa_J23119} promoter	p-P_L-s8-RybB-P_J-s28-RybB<br>p-P_L-s8-RybB-P_J-s28-MicA
GCGAAGACAA**GTAC**GATGTCC<br>CCATTTTGTGGAG	GCGAAGACAA**TTGC**GAGGGTT<br>GCAGGGTAGTAG	RybB scaffold	p-P_L-s8-RybB-P_L-s28-RybB<br>p-P_L-s8-RybB-P_J-s28-RybB<br>p-P_L-s8-RybB-P_L-s28-MicA<br>p-P_L-s8-RybB-P_J-s28-MicA
GCGAAGACAA**TGCA**GATGTCC<br>CCATTTTGTGGAG	GCGAAGACAA**ATCC**GAGGGTT<br>GCAGGGTAGTAG	RybB scaffold	p-P_L-s8-RybB-P_L-s28-RybB<br>p-P_L-s8-RybB-P_J-s28-RybB
GCGAAGACAA**TGCA**CATCCCT<br>GAATTCAGAGATG	GCGAAGACAA**ATCC**GATACCG<br>AACCGTTTGCG	MicA scaffold	p-P_L-s8-RybB-P_L-s28-MicA<br>p-P_L-s8-RybB-P_J-s28-MicA

(bold: overhangs for Golden Gate cloning; red: BbsI recognition sites)

Table S2. Oligodeoxynucleotides used for λ red recombineering, screening and Northern blot analysis.

Name	Sequence [5'-3']	Purpose
acrAB-KO-1	**ACCATTGACCAATTTGAAATCGGACACTCGAGGTTTACAT**GCTCATATGAATATCCTCCTTAG	Forward primer for deletion of *acrA* and *acrAB*
acrAB-KO-2	**AAAAAGGCCGCTTACGCGGCCTTAGTGATTACACGTTGTA**GCCTTTGAGTGAGCTGATAC	Reverse primer for deletion of *acrAB*
acrA-KO-2	**GATAAAGAAATTAGGCATGTCTTAACGGCTCCTGTTTAAG**CTAGAGCTAACTAACTTGTAGGCTG	Reverse primer for deletion of *acrA*
acrA-yfp-1	**AGCCGCAAGCGGTGCTCAGCCTGAACAGTCCAAGTCTTAA**CCGAATTCAGAGAAAGAGGAG	Forward primer for transcriptional fusion of *acrA* to *syfp2*
acrA-yfp-2	**GATAAAGAAATTAGGCATGTCTTAACGGCTCCTGTTTAAG**CTAGAGCTAACTAACTTGTAGGCTG	Reverse primer for transcriptional fusion of *acrA* to *syfp2*
acrA-yfp-5	**TCGAGGTTTACATATGAACAAAAACAGAGGGTTTACGCCT**GTTAGCAAGGGCGAAGAACTTTTTAC	Forward primer for translational fusion of *acrA* first 9 codons to *syfp2*
acrA-yfp-6	**GATAAAGAAATTAGGCATGTCTTAACGGCTCCTGTTTAAG**CTAGAGCTAACTAACTTGTAGGCTG	Reverse primer for translational fusion of *acrA* first 9 codons to *syfp2*
acrAB-scr-1	GTATGTACCATAGCACGACG	Screening of *acrA* and *acrAB* manipulations
acrAB-scr-2	GAGATCCTGAGTTGGTGG	Screening of *acrA* and *acrAB* manipulations
sYFP2_out	CGCGTCTTGTAGTTACCG	Screening of *acrA-syfp2* fusions
rybB-KO-1	**AACCGCAGAACTTTTCCGCAGGGCATCAGTCTTAATTAGT**GCTCATATGAATATCCTCCTTAG	Forward primer for deletion of *rybB*
rybB-KO-2	**GTTGAGAGGGTTGCAGGGTAGTAGATAAGTTTTAGATAAC**GCCTTTGAGTGAGCTGATAC	Reverse primer for deletion of *rybB*
rybB-scr-1	GGTATGGCCAGGATTAGG	Screening of *rybB* deletion
rybB-scr-2	GAGGATGGTTGAGAGGG	Screening of *rybB* deletion
RybB-probe-2	GAAATGGCGGGGTTGATGGGCTCCACAAAATGGGGACATC	Detection of RybB
5S probe-2	CCTGGCAGTTCCCTACTCTCGCATGAGGAG	Detection of 5S rRNA
Mult-Targ-Scr-Fw	CTGTCAAATGGACGAAGCAG	Forward primer for colony PCR of Golden Gate constructs
Mult-Targ-Scr-Rev	CAGGCAAATTCTGTTTTATCAGACC	Reverse primer for colony PCR of Golden Gate constructs

(bold: overhangs for homologous recombination; underlined: sequences for PCR amplification)
